# Secondary Metabolism and Biotrophic Lifestyle in the Tomato Pathogen *Cladosporium fulvum*

**DOI:** 10.1371/journal.pone.0085877

**Published:** 2014-01-17

**Authors:** Jérôme Collemare, Scott Griffiths, Yuichiro Iida, Mansoor Karimi Jashni, Evy Battaglia, Russell J. Cox, Pierre J. G. M. de Wit

**Affiliations:** 1 Laboratory of Phytopathology, Wageningen University, Wageningen, The Netherlands; 2 Centre for Biosystems Genomics, Wageningen, The Netherlands; 3 National Institute of Vegetable and Tea Science, Tsu, Japan; 4 Department of Plant Pathology, Tarbiat Modares University, Tehran, Iran; 5 School of Chemistry, University of Bristol, Bristol, United Kingdom; Wageningen University, The Netherlands

## Abstract

*Cladosporium fulvum* is a biotrophic fungal pathogen that causes leaf mould of tomato. Analysis of its genome suggested a high potential for production of secondary metabolites (SM), which might be harmful to plants and animals. Here, we have analysed in detail the predicted SM gene clusters of *C. fulvum* employing phylogenetic and comparative genomic approaches. Expression of the SM core genes was measured by RT-qrtPCR and produced SMs were determined by LC-MS and NMR analyses. The genome of *C. fulvum* contains six gene clusters that are conserved in other fungal species, which have undergone rearrangements and gene losses associated with the presence of transposable elements. Although being a biotroph, *C. fulvum* has the potential to produce elsinochrome and cercosporin toxins. However, the corresponding core genes are not expressed during infection of tomato. Only two core genes, *PKS6* and *NPS9*, show high expression *in planta*, but both are significantly down regulated during colonization of the mesophyll tissue. *In vitro* SM profiling detected only one major compound that was identified as cladofulvin. *PKS6* is likely involved in the production of this pigment because it is the only core gene significantly expressed under these conditions. Cladofulvin does not cause necrosis on *Solanaceae* plants and does not show any antimicrobial activity. In contrast to other biotrophic fungi that have a reduced SM production capacity, our studies on *C. fulvum* suggest that down-regulation of SM biosynthetic pathways might represent another mechanism associated with a biotrophic lifestyle.

## Introduction

Fungi are a major source of natural compounds, also known as secondary metabolites (SMs), with diverse biological activities. Fungal SMs include important pharmaceuticals such as penicillin and lovastatin, but also harmful food and feed contaminants known as mycotoxins including aflatoxins and trichothecenes. They can also serve as pathogenicity factors such as host-specific and non-specific toxins produced by many fungal plant pathogens [1], [2]. Fungal SMs are classified into four main groups based on core enzymes and precursors involved in their biosynthesis: polyketides, non-ribosomal peptides, terpenes and alkaloids [3]. In contrast to plants, fungi produce mainly polyketides and non-ribosomal peptides, and accordingly contain a higher number of core genes encoding polyketide synthases (PKSs) and non-ribosomal peptide synthetases (NRPSs) in their genomes [4]. SM biosynthetic pathways often require several enzymes that are encoded by co-regulated genes located at the same locus in the genome, which defines a gene cluster organization [5].

The genomics era has provided new tools to study fungal SMs and their biosynthesis at the whole genome scale. The core enzymes typically responsible for the synthesis of the first intermediate in biosynthetic pathways, *i.e.* PKSs, NRPSs, hybrid PKS-NRPSs, terpene cyclases (TCs) and dimethylallyl tryptophan synthase (DMATSs), have highly conserved domains which allows efficient identification of their encoding genes. From the inventory of these genes in a given genome, the SM production capacity of a fungal species can be assessed. This genome-wide approach already showed that Pezizomycotina have a greater potential for SM production than Saccharomycotina, Taphrinomycotina and Basidiomycota, with up to 58 core genes predicted for *Aspergillus terreus* [4]. It also revealed that fungal biotrophy tends to be associated with a highly restricted SM production capacity [2], [6]. Indeed, SMs with necrogenic activities might be detrimental to biotrophic fungi that need living host cells to complete their lifecycle. Comparative genomics studies can facilitate the identification of gene clusters involved in the biosynthesis of previously characterized compounds. For example, the genome of *Stagonospora nodorum* contains only one hybrid PKS-NRPS, which is likely the enzyme responsible for the production of pramanicin that displays a typical hybrid polyketide structure [4]. However, only a few SMs have been characterized for a given fungus because common laboratory growth conditions are only conducive to the production of a restricted number of SMs. Thus, most of the gene clusters identified *in silico* encode cryptic or silent pathways. In *Aspergillus* species, several genetic tools that mainly rely on global gene expression modification have led to gene cluster activation and discovery of SMs like terrequinone A, nygerone and endocrocin produced by *Aspergillus nidulans*, *Aspergillus niger* and *Aspergillus fumigatus*, respectively [7]–[9]. Prior to experimental studies, phylogenetic and comparative genomics analyses are very informative as the number of fungal genomes and characterized SM pathways increases. *In silico* studies can provide new information about the organization of conserved gene clusters, their borders and evolution. Such approaches are very helpful to identify gene clusters that are involved in the production of SMs that have been characterized in other fungal species and allow subsequent predictions of identical or related compounds that a particular fungal species might produce.

*Cladosporium fulvum* is a Dothideomycete fungus responsible for tomato leaf mould disease worldwide. This fungus is a biotroph that only colonizes the apoplastic space of tomato leaves [10]. The genome of *C. fulvum* has been sequenced and bioinformatic analyses revealed that it contains 23 SM core genes, an exceptionally high number for a biotroph [11]. This finding questioned the proposed correlation between restricted SM production capacity and fungal biotrophy. In this study, we have analysed the full manifest of SM biosynthetic gene clusters in *C. fulvum* and link these to actual production of SMs and their putative role in pathogenicity. This is the first thorough study of fungal secondary metabolism that provides new insights into SM gene cluster evolution in the context of fungal biotrophy.

## Materials and Methods

### Fungal growth conditions

The sequenced strain race 0WU was grown and conidia suspensions (5×10^5^ conidia.mL^−1^) were prepared as previously described [11]. For SM detection, 500 mL of B5 medium (Gamborg B5 medium supplemented with 20 g.L^−1^ sucrose; Duchefa Biochemie B.V., The Netherlands) or PDB (Sigma-Aldrich, Saint-Louis, MO) were inoculated with 5 mL of conidia suspension and grown as shaking culture (200 rpm) for ten days at 20°C. For expression analysis, 50 mL of PDB were inoculated with 0.5 mL of conidia suspension and grown as shaking culture for seven days. Subsequently, mycelium was harvested by filtration over Miracloth, washed once with fresh medium (PDB, B5 (pH4), B5 adjusted to pH7, B5 without carbon source, B5 without nitrogen source or B5 without FeSO_4_) and transferred to flasks containing 50 mL of the corresponding medium for 48 h. Alternatively, 50 mL of B5 medium were inoculated with 0.5 mL of conidia suspensions and incubated for 12 days. Mycelium was harvested by filtration over Miracloth. Both mycelium and culture filtrate were freeze-dried. Twenty-five grams of freshly picked four-week old tomato leaves were autoclaved in flasks, inoculated with 5 mL of conidia suspension and incubated for 7 days. Taking off the leaf epidermis retrieved fungal biomass.

### Tomato inoculation and collection of apoplastic fluid

Four-week-old Heinz tomato plants, grown under standard greenhouse conditions, were sprayed with a conidia suspension (10 mL at 5×10^5^ conidia.mL^−1^) as previously described [12]. Apoplastic fluids were collected at 10 days post-inoculation following the protocol of de Wit and Spikman [13].

### Functional annotation of secondary metabolism genes

The predicted protein sequence of core enzymes was used to perform a BlastP search in the NCBI non-redundant protein database (www.ncbi.nlm.nih.gov). Conserved domains were identified using InterproScan (www.ebi.ac.uk), the PKS/NRPS analysis website [14] and ASMPKS (gate.smallsoft.co.kr:8008/∼hstae/asmpks/pks_prediction.pl; SmallSoft Co. Ltd). For each core gene, the genomic locus was inspected for upstream and downstream genes. BlastP and InterproScan analyses confirmed functional annotation of flanking genes. Borders of the gene clusters were defined when three consecutive genes did not encode proteins with a predicted function associated with secondary metabolism, or when two annotated genes were separated by more than 5 kb.

### Phylogenetic analysis

Amino acid sequences (Table S1 in File S1) of full length PKSs, KS and AT domains of hybrid PKS-NRPSs and A domains of NRPSs were aligned using T-Coffee [15]. Alignments were manually edited in Genedoc (www.psc.edu/biomed/genedoc) and poorly aligned regions were removed with Gblocks, allowing smaller final blocks, gap positions within the final blocks and less strict flanking positions [16]. Phylogenetic trees were constructed using the maximum likelihood algorithm with default parameters apart from the JTT amino acid substitution matrix and were edited in MEGA5 [17].

### Gene expression analysis

Total RNA from infected tomato leaves was already available [11]. New biological repeats were performed following the same protocol and the same methods were used to extract total RNA from mycelium grown under *in vitro* conditions. As a negative control, RNA was also extracted from healthy plants. cDNA synthesis was performed using 2 µg of total RNA and the First Strand cDNA synthesis kit (Invitrogen, Carlsbad, CA) or M-MLV reverse transcriptase (Promega, Madison, WI), following the manufacturer's protocol. For quantitative PCR, primers were designed with Primer3Plus [18] (Table S2 in File S1) and their efficiency was tested on a genomic DNA dilution series. Quantitative PCR was performed with the Applied Biosystems 7300 Real-Time PCR System (Applied Biosystems, USA). Raw data were analysed following the 2^−ΔCt^ method [19]. Expression of the actin gene was used to normalize the expression of each gene. This normalization was assessed using the β-tubulin gene as a control. Results are average of two to three biological repeats, and up to two technical repeats.

### Clamped homogenous electric fields (CHEF) gel electrophoresis and Southern blotting

*C. fulvum* protoplasts were prepared as previously described [20], using 5 mg.mL^−1^ Kitalase (Wako Pure Chemicals) and 10 mg.mL^−1^ Lysing Enzyme (Sigma-Aldrich, Saint-Louis, MO), incubating mycelium overnight at 30°C with gentle shaking. Chromosome-sized DNA molecules were extracted as previously described [20], and separated in 0.8% agarose gels (SeaKem Gold Agarose; Lonza, Rockland, ME) at 8°C using 0.5× TBE buffer. CHEF electrophoresis was carried out with a CHEF-DRII apparatus (Bio-Rad, Hercules, CA) with the following settings (duration/voltage/linear gradient of switching time): 115 hrs/50 V/3600–1800 sec; 24 hrs/50 V/1800–1300 sec; 28 hrs/60 V/1300–800 sec; 28 hrs/80 V/800–600 sec. The gel was stained with ethidium bromide for 30 min. DNA gel blotting was performed using standard methods [21]. DNA was transferred onto Hybond N+ nylon membranes (GE Healthcare, Little Chalfont, UK) and subjected to hybridization and detection using DIG High Prime DNA Labelling and Detection Starter Kit II (Roche, Basel, CH) according to the manufacturer's instructions.

### Secondary metabolite extraction and thin layer chromatography

Freeze-dried culture filtrate and mycelium were extracted twice in 25 mL of ethyl acetate, incubating the samples at room temperature for 10 min in an ultrasound bath, then shaking for 30 min in a hood. The organic phase was collected by centrifugation for 10 min at 4,000×g and evaporated under nitrogen flow. Concentrated extracts dissolved in acetonitrile were spotted on TLC Silica Gel 60 plates (Merck Millipore, Billerica, MA). The mobile phase was composed of toluene/ethyl acetate/formic acid (5∶4∶1; v/v/v). Fluorescent compounds were detected at 254 nm and 365 nm wavelengths using a ChemiDoc™ XRS+ System (Bio-Rad) and a Black-Ray® long wave UV lamp model B 100AP (Mineralogical Research Co., San Jose, CA), respectively. Plates were stained using iodine vapour from crystals in a closed tank. Cladofulvin was located by spraying the plates with magnesium acetate (4% w/v in MeOH).

### Purification and characterisation of cladofulvin

Extracts of *C. fulvum* were purified using a mass-directed chromatography system consisting of a Waters 2767 auto-sampler, Waters 2545 pump system, Phenomenex LUNA column (5μ, C_18_, 100 Å, 10×250 mm) equipped with a Phenomenex Security Guard pre-column (Luna C_5_ 300 Å) eluted at 4 mL⋅min^−1^. Solvent **A**, HPLC grade H_2_O+0.05% formic acid; Solvent **B**, HPLC grade MeOH+0.045% formic acid; solvent **C**, HPLC grade CH_3_CN+0.045% formic acid. The post-column flow was split (100∶1) and the minority flow was made up with solvent **B** to 1 mL⋅min^−1^ for simultaneous analysis by diode array detector (Waters 2998), evaporative light scattering (Waters 2424) and ESI mass spectrometry in positive and negative modes (Waters Quatro Micro). The following chromatographic programme was used (the balance of solvent used was **A**): 0 min, 20% **C**; 10 min 90% **C**; 26 min 95% **C**; 28.5 min 95% **C**; 29 min 20% **C**; 30 min 20% **C**. Fractions containing cladofulvin were combined and evaporated to afford 2.2 mg of a bright orange solid. *m/z* (ES^+^) 539.1 [M]H^+^, 561.1 [M]Na^+^; (ES^−^) 537.2 [M−H]^−^, 559.2 [M−2H+Na]^−^. uv max (CH_3_CN) 235.1, 269.1. ^1^H NMR: 500 MHz, CD_3_OD/CD_3_CN; ^13^C NMR: 125 MHz, CD_3_OD/CD_3_CN (Table S3 in File S1). HRMS, calc for C_30_H_18_O_10_ 537.0822, observed 537.0801 [M−H]^−^.

### Biological activity assays

Purified cladofulvin was solubilised in CH_3_CN to 10 mM. Dilution series of pure cladofulvin were applied onto paper discs displayed on LB or PDA plates. CH_3_CN (10% v/v in water) was used as negative control. An agar overlay was immediately poured, containing either cells of *Pseudomonas fluorescens*, *Streptomyces coelicolor* (OD_600_ = 1), or spores of *Botrytis cinerea* B05.10 strain or *Hansfordia pulvinata* (10^4^ spores). Plates were incubated for two days at 25°C for *P. fluorescens* and at 30°C for *S. coelicolor*. Plates were incubated for eight days at 18°C and 22°C for *B. cinerea* and *H. pulvinata*, respectively. The same diluted samples were infiltrated in leaves of *Solanum lycopersicum* and *Nicotiana benthamiana* plants. Infiltrated leaves were monitored for seven days. Each experiment was performed under light and dark conditions at least twice.

## Results

### Inventory of *Cladosporium fulvum* secondary metabolism core genes

The genome of *C. fulvum* contains 23 core SM genes (10 PKSs, 10 NRPSs, 2 hybrid PKS-NRPSs and 1 DMATS, no TC). Of these, 15 are likely to encode functional enzymes, but seven appear to be pseudogenes and/or truncated genes (*PKS4*, *PKS9*, *NPS1*, *NPS5*, *NPS7*, *NPS10* and *HPS2*) [11]. Five PKSs (PksA, Pks1, Pks6, Pks7 and Pks9) show the typical organization of non-reducing PKSs (starter unit (SAT)-keto synthase (KS)-acyl transferase (AT)-product template (PT)-acyl carrier protein (ACP) domains) (Figure S1 and Table S4 in File S1), suggesting that they produce aromatic compounds [22]. The other five PKSs share the organization of partially- and highly-reducing PKSs with the additional DH-(ER)-KR (dehydratase-(enoyl reductase)-keto reductase) domains, suggesting that they may produce reduced (possibly linear) polyketide chains [22]. PksA, Pks1 and Pks7 contain a TE domain possibly involved in the release of the polyketide chain (Figure S1 in File S1) [23]. The other PKSs do not appear to contain thiolesterase (TE) domains, suggesting other mechanisms for product release [23].

Of the NRPSs, six are mono/bi-modular enzymes (Nps1, Nps4, Nps5, Nps6, Nps8 and Nps10), while Nps2, Nps3, Nps7 and Nps9 are multi-modular enzymes (Figure S1 in File S1). However, pseudogenes or truncated genes encode half of them. All predicted functional NRPSs have a terminal condensation (C) domain that might be involved in the release of the peptide from the enzyme [24]. Nps2 shows the typical organization of type IV fungal siderophore synthetases and Nps9 that of type II enzymes (Table S4 in File S1) [25], [26]. Signature specificities of the adenylation (A) domains of these two synthetases are consistent with siderophore biosynthesis.

### Phylogenetic analysis of *Cladosporium fulvum* secondary metabolism core enzymes

**Figure 1:**
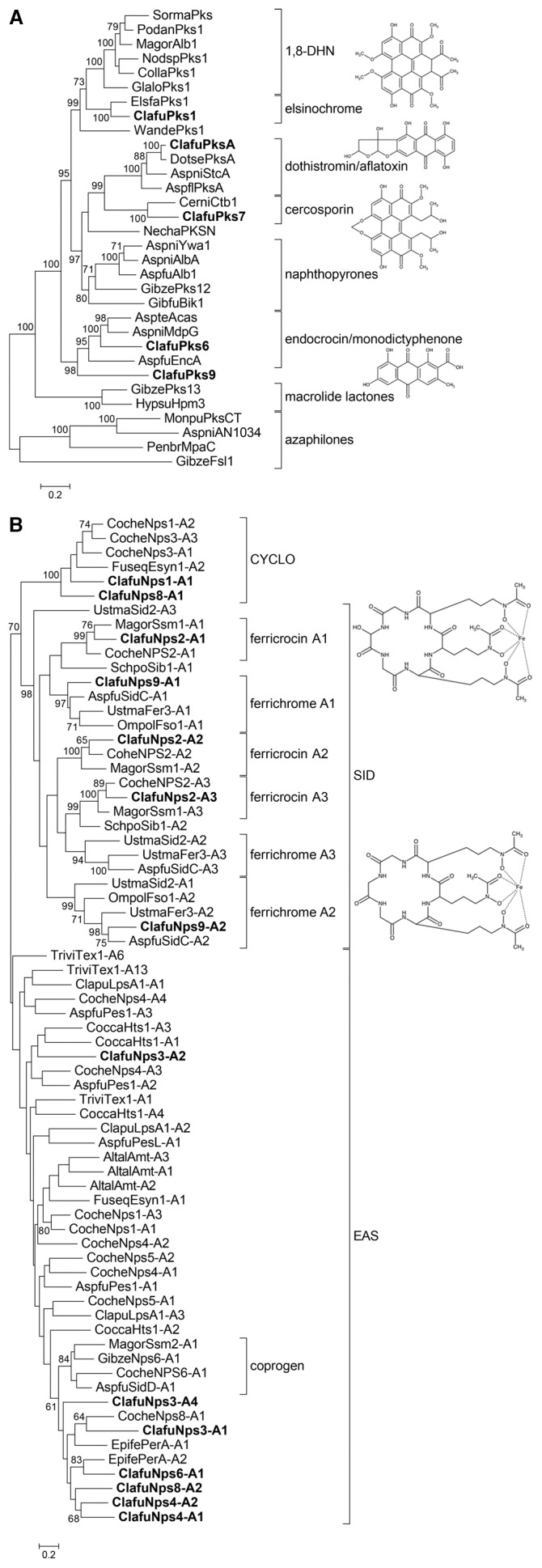
Phylogenetic analysis of *Cladosporium fulvum* PKS and NRPS enzymes. Alignments of (**A**) full-length protein sequences of non-reducing polyketide synthases (PKSs) and (**B**) A-domains of non-ribosomal peptide synthetases (NRPSs) were used to construct maximum likelihood phylogenetic trees. The third A domain of ClafuNps9 was not included because of poor alignment. CYCLO: cyclosporine synthetases; SID: siderophore synthetases; EAS: Euascomycete clade synthetases. Only bootstrap values over 70 are shown. *C. fulvum* secondary metabolism enzymes are indicated in bold. The chemical structures of secondary metabolites potentially produced by *C. fulvum* are shown. Accession numbers are given in Table S1 and Table S4 in File S1.

Phylogenetic analyses that resolve orthologous relationships can predict the structural type of SM that will be synthesized by a given conserved core enzyme [27]–[29]. Phylogenetic trees were constructed using deduced amino acid sequences of core proteins characterized in other fungal species (*i.e.* they were shown to be involved in the biosynthesis of characterized SMs), of enzymes representing conserved phylogenetic clades and of those of *C. fulvum* that are not truncated. Sequences of fungal non-reducing PKSs, reducing PKSs, and the KS and AT domains of hybrid PKS-NRPSs were aligned to construct the respective maximum likelihood phylogenetic trees. Three out of the five non-reducing PKSs are orthologues of characterized enzymes (PksA, Pks1 and Pks7; Figure 1A). It was previously reported that PksA is related to the dothistromin synthase of *Dothistroma septosporum*, which is itself orthologous to synthases that are responsible for the biosynthesis of the highly toxic aflatoxins and sterigmatocystins [11]. Pks1 and Pks7 are orthologues of the elsinochrome synthase EfPKS1 from *Elsinoë fawcettii* [30] and the cercosporin synthase CTB1 from *Cercospora nicotianae* [31], respectively (Figure 1A). The two remaining non-reducing PKSs, Pks6 and Pks9, belong to a clade that comprises synthases responsible for the production of atrochrysone [32], monodictyphenone [33] and endocrocin [9].

The phylogenetic tree for reducing PKSs shows that Pks2 belongs to a clade that includes Dep5 and BCBO9, which are involved in the production of depudecin and botcinic acid, respectively (Figure S2A in File S1) [34], [35]. Because the biosynthesis of botcinic acid requires a second PKS that is not present in *C. fulvum*, it is likely that Pks2 produces a compound with a backbone related to depudecin. Pks3 and Pks5 are closely related to the alternapyrone synthase, PksN, of *Alternaria solani* and the T-toxin synthase, Pks2, of *Cochliobolus heterostrophus*, respectively [36], [37]. In contrast, Pks8 is not related to any characterized PKS. The only functional PKS-NRPS hybrid in *C. fulvum*, Hps1, belongs to a clade that includes hybrids involved in cyclopiazonic acid biosynthesis (Figure S2B in File S1) [38].

The A-domain sequences of *C. fulvum* NRPS enzymes could be aligned with A-domains sequences representative of the different NRPS classes described by Bushley and Turgeon [39]. The phylogenetic tree shows that *C. fulvum* NRPS A domains group to three clades only: SID (siderophore synthetases), EAS (Euascomycete clade synthetases) and CYCLO (cyclosporin synthetases) (Figure 1B). Nps1 belongs the CYCLO clade and Nps8 is a hybrid protein containing A domains that belong to the CYCLO and EAS clades. Nps3, Nps4 and Nps6 belong to the EAS clade. Several NRPSs of the CYCLO clade are recombinant proteins containing an A-domain that belongs to the EAS clade [39]. This suggests that *NPS1* might be a truncated gene, which is consistent with its unusual domain organization. The Nps6 sole A-domain is related to the peramine synthetase of *Epichloë festucae*. Both enzymes carry a *N*-MeT domain, but the PerA synthetase is bi-modular, while Nps6 is mono-modular [40]. Similar to *NPS1*, *NPS6* could also be a truncated gene. Nps2 and Nps9 A-domains are closely related to cognate A-domains of synthetases responsible for the biosynthesis of siderophores of the ferricrocin and ferrichrome type (Figure 1B).

Although this analysis predicts the type of SMs that might be produced by *C. fulvum* (reduced/non-reduced polyketides, siderophores), it does not provide any information on the precise chemical structure.

### Rearrangements in secondary metabolism gene clusters in the *Cladosporium fulvum* genome

Fifteen putative SM gene clusters have been identified in the *C. fulvum* genome (Figure S3 and S4 in File S1). Five core genes (two pseudogenes and one likely truncated gene) do not belong to any predicted gene cluster (*PKS5*, *HPS1*, *HPS2*, *NPS1* and *NPS7*), but they could have been part of gene clusters that have been disrupted by transposable elements, which are abundant in the *C. fulvum* genome [12]. The *PKSA* gene cluster has already been described in detail and is homologous to the complete dothistromin biosynthetic gene cluster in *D. septosporum* [12]. However in *C. fulvum*, *HEXA* and *NOR1* are pseudogenes, resulting in a non-functional pathway [41]. Indeed, these two genes encode enzymes responsible for the synthesis of the first (hexanoate) and third (averantin) intermediates in the dothistromin biosynthetic pathway. In *D. septosporum*, this cluster is fragmented over six loci on chromosome 12 [42]. In *C. fulvum*, three loci are located on a small chromosome of 0.8 Mb and the other three loci are located on a 1.8 Mb chromosome (Figure S5 in File S1), suggesting that inter-chromosomal rearrangements further disrupted the putative ancestral dothistromin cluster in this fungus.

The organization of all other SM loci in the *C. fulvum* genome is fully depicted (Figure S3 and S4 in File S1). All 15 predicted gene clusters contain genes that encode typical accessory enzymes including dehydrogenases, cytochrome P450 mono-oxygenases and methyltransferases. Several clusters also comprise MSF/ABC transporter and transcription factor encoding genes that might be involved in SM secretion and local gene cluster regulation, respectively. Small chromosomes do not tend to be enriched in SM gene clusters in *C. fulvum* because *PKS6* is located on a 4 Mb chromosome and both *PKS1* and *NPS9* are found on the same 2.9 Mb chromosome, while *PKS7* is located on a 1.8 Mb chromosome (Figure S5 in File S1).

All orthologues of *C. fulvum* core genes belong to predicted or characterized gene clusters in other fungal species. However in *C. fulvum*, no conserved gene clusters could be found at the *PKS2*, *PKS3*, *PKS5*, *PKS9* and *NPS6* loci. This suggests that these pathways are involved in the biosynthesis of SMs that differ from those identified in other fungi. Thus, it is unlikely that *C. fulvum* can produce depudecin, alternapyrone, T-toxin or peramine. In contrast, conserved gene clusters were identified at the *PKS1*, *PKS6*, *PKS7*, *NPS2* and *NPS9* loci, which are responsible in other fungi for the biosynthesis of elsinochrome, monodictyphenone, cercosporin and siderophore production, respectively.

**Figure 2:**
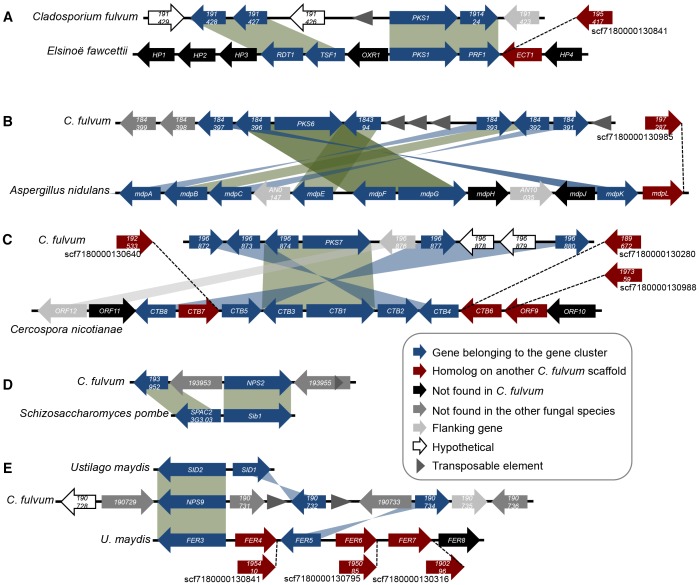
Synteny and rearrangements of conserved secondary metabolism gene clusters in *Cladosporium fulvum*. The organization of gene clusters conserved in *C. fulvum* was compared to the previously described clusters involved in the biosynthesis of (**A**) elsinochrome, (**B**) monodictyphenone, (**C**) cercosporin, (**D**) ferricrocin and (**E**) ferrichrome in other fungi. Genes are represented as arrows, indicating their orientation. Representation of genes is not to scale.

The gene cluster for elsinochrome biosynthesis in *E. fawcettii* comprises six genes (*PKS1*, *PRF1*, *RDT1*, *TSF1*, *ECT1* and *OXR1*) [43]. Four additional flanking genes (*EfHP1* to *EfHP4*) that encode hypothetical proteins are located at the same locus, but none are present in the genome of *C. fulvum* (Figure 2A). The elsinochrome gene cluster organization shows conserved synteny between both species for all genes, but in *C. fulvum OXR1* has been replaced by a transposable element and a gene encoding a hypothetical protein (Figure 2A). Transposon insertion at this locus could have resulted in the loss of *OXR1* and insertion of another gene in *C. fulvum*. A homologue of *ECT1* is present on another scaffold in *C. fulvum*, suggesting that rearrangements might have occurred at the border of this gene cluster.

Monodictyphenone, atrochrysone and endocrocin share a similar biosynthetic pathway that requires conserved PKS and β-lactamase-type thiolesterase enzymes [9], [32], [33]. The *PKS6* gene cluster contains seven genes that are part of the monodictyphenone gene cluster in *A. nidulans* (Figure 2B) [33]. This cluster in *C. fulvum* shows rearrangements associated with the presence of many transposable elements. Remarkably, genes located within the monodictyphenone gene cluster but not involved in the biosynthesis of this SM are all lost in *C. fulvum*. In addition, *mdpH* and *mdpJ* are also absent, while an *mdpL* homologue is found on another scaffold. The *PKS6* gene cluster comprises two additional genes that putatively encode a cytochrome P450 mono-oxygenase and a dehydrogenase (Figure 2B).

*PKS7* is an orthologue of the cercosporin synthase gene, *CTB1*, in *C. nicotianae*, which belongs to a gene cluster of eight genes (*CTB1* to *CTB8*), flanked by genes with a different transcriptional regulation (*ORF9* to *ORF12*) [44]. Homologues of six genes from the cercosporin gene cluster are present at the *PKS7* locus in *C. fulvum*. *PKS7/CTB1* and *CTB3* form a core set of genes and rearrangements occurred on both sides in *C. fulvum* (Figure 2C). Although *CTB6* and *CTB7* are not present at this locus, homologues were identified on different scaffolds. Despite extensive rearrangements, there is no evidence for transposon activity that could be associated with rearrangements at this locus.

The biosynthesis of ferricrocin requires two genes that are located at the same locus in the genome of *Schizosaccharomyces pombe*, which encode an L-ornithine N5-oxygenase and an NRPS [25]. Similarly, in *C. fulvum*, a homologue of the L-ornithine N5-oxygenase gene is located next to *NPS2* (Figure 2D). In addition, a gene encoding an ABC transporter that was either inserted in *C. fulvum* or lost in *S. pombe* is located in between these two genes.

The biosynthesis of ferrichrome siderophores has been extensively studied in the Basidiomycota *Ustilago maydis*. Sid1 is an L-ornithine N5-oxygenase involved in the production of both ferrichrome and ferrichrome-A [45], while Sid2 and Fer3 are NRPSs involved in the production of ferrichrome and ferrichrome-A, respectively [45], [46]. In *U. maydis*, *SID1* and *SID2* are located at the same locus and *FER3* belongs to another gene cluster that comprises six genes (*FER3* to *FER8*) required for ferrichrome-A biosynthesis. The *NPS9* gene cluster identified in *C. fulvum* seems to be a combination of both loci present in *U. maydis* because it contains homologues of *SID1* and *FER5* in addition to the NRPS gene (Figure 2E). Other homologous genes of the *FER3* gene cluster are present scattered on different scaffolds in *C. fulvum*. Likely, siderophores produced by *C. fulvum* are different from those produced by *U. maydis*, but they likely can sequester iron. Indeed, differences in NRPS post-assembly steps will result in varied compounds that are still functional siderophores as exemplified by those of the ferrichrome family [47].

### Most SM functional core genes are weakly expressed or down-regulated during colonization of tomato leaves

**Figure 3:**
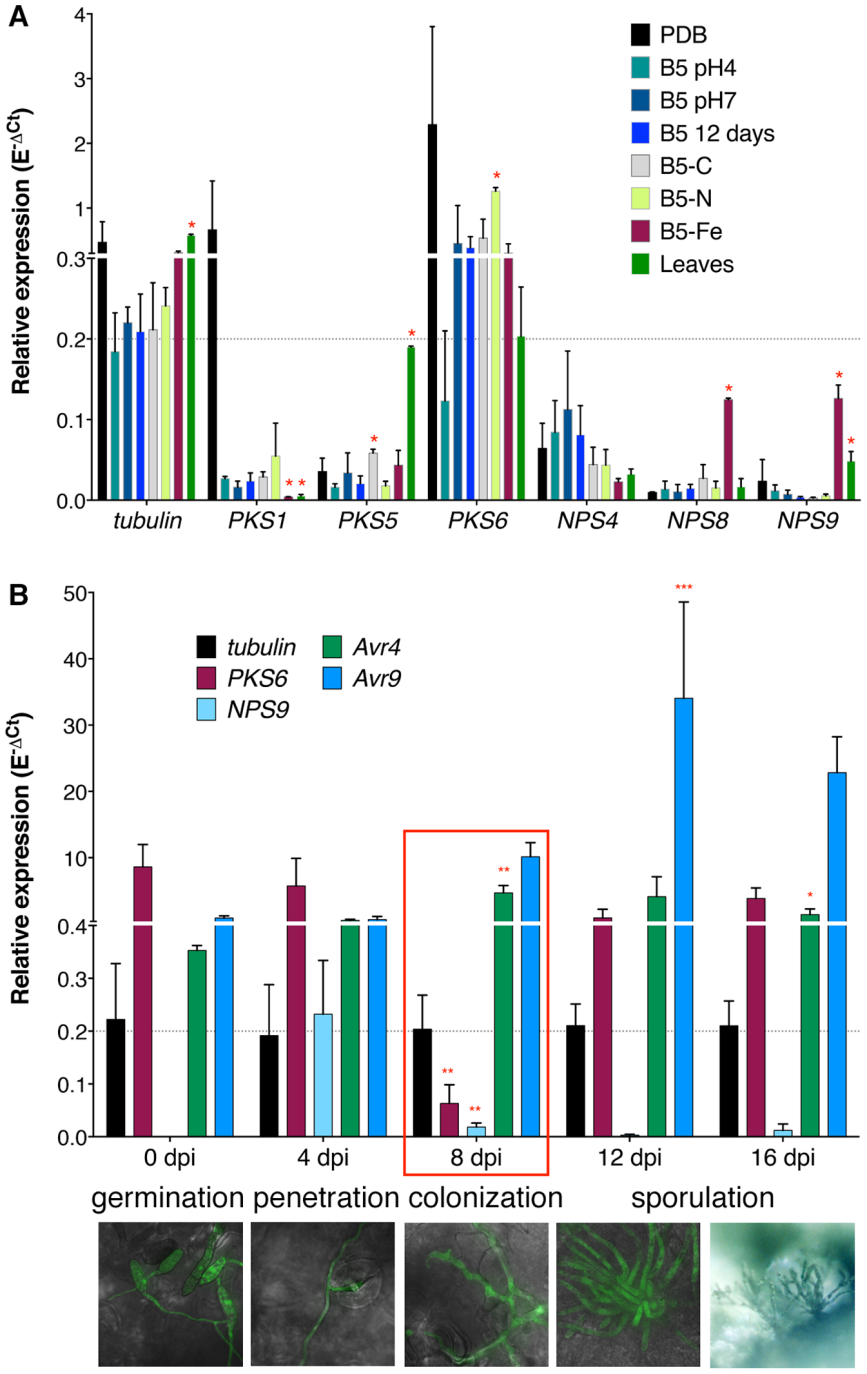
Expression profile of *Cladosporium fulvum* secondary metabolism functional core genes. Expression profiles were measured by RT-qrtPCR using RNA isolated from mycelium grown in diverse *in vitro* growth conditions: PDB, B5 with different pHs, B5 without carbon source (B5-C), B5 without nitrogen source (B5-N), B5 without FeCl_3_ (B5-Fe), stationary phase (B5-12days) and autoclaved tomato leaves; and from tomato plants inoculated with the sequenced *C. fulvum* race 0WU strain from 0 to 16 days post-inoculation (dpi). Results are shown relative to the actin gene expression according to the E^−ΔCt^ method, where E is the efficiency of a given primer pair. Tubulin gene was used as a control for calibration and the effector genes *Avr4* and *Avr9* were used as positive controls for the tomato infection experiment. The grey dotted line indicates the tubulin expression level. Values are the mean of three biological repeats and the error bars represent standard deviations (SD). (**A**) Only six genes show expression during *in vitro* growth, while (**B**) two genes are down-regulated during colonization of tomato. Pictures of tomato infected by a GFP-tagged *C. fulvum* strain are shown below to indicate the development of the fungus at the different time points of infection. Expression in each *in vitro* condition was compared to that in B5 pH4 medium using multiple t-tests, not assuming consistent SD, correcting for multiple comparisons with the Holm-Sidak method. For each gene, each *in planta* time point was compared to the previous one using a Two-way ANOVA followed by a multiple comparison test corrected with the Holm-Sidak method. All statistical tests were performed at the alpha significance threshold of 0.05. Red asterisks indicate significant differences only (* p<0.01; ** p<0.001; *** p<0.0001).

To further characterize the functional core genes, expression analysis was performed under eight different *in vitro* growth conditions and during infection of tomato using Reverse Transcription quantitative real-time PCR (RT-qrtPCR). Only *PKS6* exhibited higher expression than the tubulin gene in almost all *in vitro* conditions tested (Figure 3A). *PKS1* was highly expressed in PDB medium only, *NPS4* exhibited clear expression in all conditions, and *PKS5* and *NPS9* genes were induced when *C. fulvum* was grown on autoclaved leaves. *NPS8* and *NPS9* were significantly induced under iron depletion, which is consistent with the prediction that Nps9 is a siderophore synthetase. All other genes exhibited a very low expression level that is about ten times below that of the tubulin gene (Figure S6A in File S1). During tomato infection (from 0 days post-inoculation (dpi) to 16 dpi), only *PKS6* and *NPS9* exhibited high expression at the early stages of infection when runner hyphae were growing on the leaf surface (Figure 3B). Remarkably, their expression dropped after penetration when the fungus was colonizing the apoplastic space between mesophyll cells. The expression of *PKS6* was induced at later stages of infection when conidiophores emerged from the plant and produced conidia. A similar expression profile was observed for most genes located in the predicted *PKS6* gene cluster, except for two of them whose expression was only detected from 12 or 16 dpi (Figure S7 in File S1). These genes encode homologues of the mdpB reductase and mdpE transcription factor of the monodictyphenone pathway. In contrast, the effector genes *Avr4* and *Avr9* were strongly induced during colonization of tomato leaves (Figure 3B). Expression of all other core genes remained low or even barely detectable during the whole infection cycle (Figure S6B in File S1), although a few genes might show weak down- or up-regulation (Figure S6C and S6D in File S1). Thus, it appears that most of the potentially functional SM pathways are silent under the tested *in vitro* conditions and only show very low expression when the fungus is growing inside its host.

### Production of secondary metabolites by *Cladosporium fulvum*

**Figure 4:**
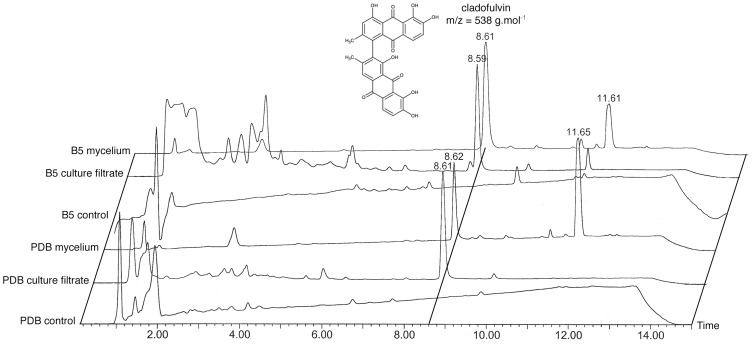
*Cladosporium fulvum* secondary metabolite profiling. Secondary metabolites (SMs) were extracted from *in vitro* liquid cultures in PDB and B5 media. Non-inoculated media served as negative controls. Culture filtrate and mycelium were separated by filtration prior to SM extraction and LC-MS analysis. Cladofulvin (retention time ca. 8.6 min) was the only SM detected in these samples. The peak at ca. 11.6 min corresponds to the fatty acid linoleic acid.

The present combined phylogenetic and comparative genomics analyses suggest that *C. fulvum* has the genetic potential to produce at least two polyketides (elsinochrome- and cercosporin-related compounds) and two non-ribosomal peptides (ferricrocin- and ferrichrome-related siderophores). In addition, *C. fulvum* was shown to produce the bianthraquinone pigment cladofulvin [48]. Production of these compounds by the sequenced wild type race 0WU strain was assessed under different *in vitro* conditions (12 days in PDB and B5 media). Culture filtrates and mycelium were separated by filtration prior to extraction and thin layer chromatography (TLC) and liquid chromatography/mass spectrometry (LC-MS) analyses. LC-MS profiling revealed that mycelium produced only two abundant metabolites that eluted at ca. 8.60 and 11.60 minutes, respectively (Figure 4). The first LC-MS peak corresponds to a compound with a mass *m/z* of 538 g.mol^−1^, which is the molecular weight of cladofulvin. This SM is secreted because it is also detected in the culture filtrate. It corresponds to the sole yellow spot observed on silica TLC plates viewed under visible light, which turns into violet when treated with magnesium acetate as previously reported for cladofulvin (Figure S8 in File S1) [48]. Nuclear magnetic resonance (NMR) analysis confirmed the structure of cladofulvin after purification of the corresponding LC-MS peak (Table S3 in File S1). The second LC-MS peak was identified as linoleic acid, a common unsaturated fatty acid, based on the fragmentation spectrum and UV absorbance. Extracts from culture filtrate contain additional early-eluted peaks for which no *m/z* could be assigned. Accordingly, TLC analysis revealed the presence of many additional compounds when viewed under UV light and after iodine staining (Figure S8 in File S1). Metabolites were also extracted from apoplastic fluids collected from healthy and infected tomato plants, but the metabolite concentration in these samples did not allow assigning any mass and subsequent identification (data not shown).

Toxicity of the purified cladofulvin was tested on *Solanaceae* plants (*Solanum lycopersicum* and *Nicotiana benthamiana*) grown under light and dark conditions. While the positive control dothistromin triggered light-dependent necrosis (toxicity was much weaker on tomato), cladofulvin did not induce any necrosis (Figure S9 in File S1), even at concentrations in the mM range (data not shown). Cladofulvin was also tested for toxicity against bacteria (*Pseudomonas fluorescens* and *Streptomyces coelicolor*) and fungi (*Botrytis cinerea* and *Hansfordia pulvinata*, a fungal antagonist of *C. fulvum*), but no antimicrobial activity against any of these organisms was observed (data not shown). The biological role of cladofulvin is unlikely related to pathogenicity or colonization of an ecological niche. Consistent with only cladofulvin being produced by *C. fulvum in vitro*, crude organic extracts from mycelium or culture filtrate did not show any antimicrobial activity against all these organisms grown under light or dark conditions (data not shown).

## Discussion

### Transposable elements have shaped the *Cladosporium fulvum* secondary metabolome

Loss of SM clusters has been suggested to possibly involve presence/activity of transposable elements. Yet, clear evidence is still missing. For example, *Sclerotinia sclerotiorum* and *B. cinera* are closely related fungi, with *S. sclerotiorum* containing more transposons in its genome [49]. The latter species has lost several SM gene clusters that are present in *B. cinerea*, but these losses are not associated with transposable elements. In contrast, comparison of all SM loci between *C. fulvum* and *D. septosporum* suggested transposable element-mediated gene losses in *C. fulvum* [12]. The present study also suggests that transposition events might have shaped the gene content and organization of SM gene clusters in *C. fulvum*. Indeed, all core genes except *PKSA* and *PKS7* are located at loci that contain at least one transposable element. Moreover, five gene clusters are located at scaffold borders, which are composed of multiple repeats, and five other gene clusters reside on small scaffolds that contain no other genes.

**Figure 5:**
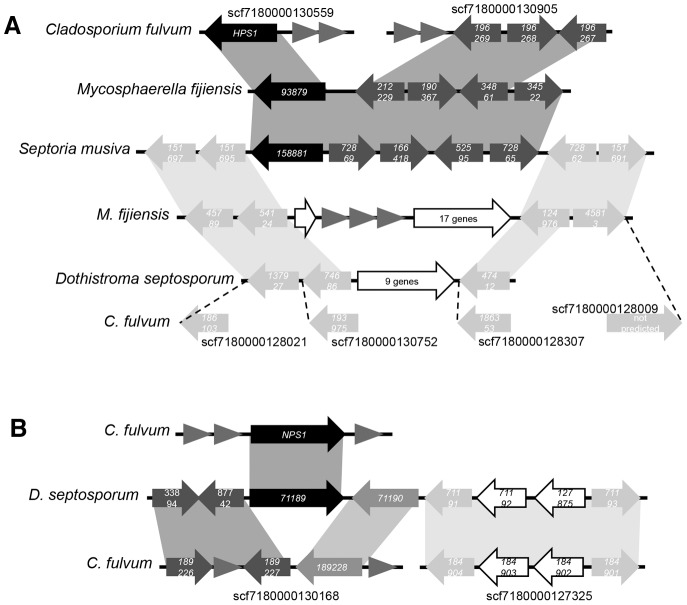
Disruption of *Cladosporium fulvum* gene clusters by transposable elements. (**A**) The *HPS1* and (**B**) *NPS1* genes in *C. fulvum* are orthologs of core genes that belong to conserved gene clusters in related fungal species. Genes are represented as arrows, indicating their orientation. Black arrows are genes with conserved orientation. Dark grey arrows are genes in the gene clusters that have undergone rearrangements. Light grey arrows are genes that border the gene cluster. Triangles represent transposable elements. Representation of genes is not to scale.

The *C. fulvum* genome contains at least two striking examples of gene cluster disruption that is likely the result of transposon activity. *HPS1* and *NPS1* do not belong to gene clusters and they are located on small scaffolds delimited by many repeats. However, orthologues of these two core genes were found in related fungal species. *HPS1* orthologues in *Mycosphaerella fijiensis* and *Septoria musiva* are part of a conserved gene cluster that contains four additional genes (Figure 5A). Interestingly, homologues of three of these genes are located at a single locus on another scaffold in *C. fulvum*. This suggests that in *C. fulvum HPS1* was also part of this conserved gene cluster. Moreover, the gene cluster borders in *S. musiva* are conserved at a different locus in *M. fijiensis* and *D. septosporum*, while they are scattered over different scaffolds in *C. fulvum*. The gene content in between these border genes in *M. fijiensis* and *D. septosporum* is different. This observation suggests that this conserved locus is prone to rearrangements, which might have led to the loss of the *HPS1* gene cluster in *D. septosporum* and to its re-localization to another locus in *M. fijiensis*. *NPS1* has an orthologue in *D. septosporum* where it belongs to a predicted gene cluster (Figure 5B). The other genes from this gene cluster are located at two different loci in the *C. fulvum* genome. Similarly to *HPS1*, it is likely that transposable elements led to rearrangements in this cluster and to the re-localization of *NPS1* to another locus. These examples show that *C. fulvum* gene clusters suffered from severe rearrangements likely due to transposon activity, which may have led to inactivation of several SM biosynthetic pathways. Remarkably, the conserved gene clusters that might be involved in the biosynthesis of elsinochrome-, cercosporin- and ferricrocin-like compounds are not associated with many transposable elements and are located in the middle of scaffolds, although some rearrangements have occurred. Retention of these gene clusters might point to an important role of their products at specific stages of the *C. fulvum* lifecycle.

### Prediction of a fungal secondary metabolome

In this study, we made use of phylogenetics and comparative genomics analyses to predict SMs that *C. fulvum* might produce. All genes involved in the dothistromin biosynthetic pathway present in *D. septosporum* have homologues located on two chromosomes in *C. fulvum*. The *PKS7* gene cluster is homologous to the cercosporin gene cluster in *C. nicotianae*. However, both *PKSA* and *PKS7* are among the core genes with the lowest expression level under all conditions tested. In addition, homologues of several genes required for early steps in dothistromin biosynthesis are pseudogenes in *C. fulvum* [41]. Accordingly, TLC and LC-MS analyses could detect neither dothistromin nor cercosporin under the growth conditions tested.

It is hypothesized that plant pathogenic fungi secrete siderophores for iron acquisition from the external environment during the infection process and synthesize intracellular siderophores for iron storage [50]. Ferricrocin was reported to be an intracellular siderophore in *S. pombe* and *Magnaporthe oryzae* [51], in which no transporter has been reported at the NRPS gene locus. *NPS2* and *NPS9* belong to gene clusters predicted to be involved in the biosynthesis of ferricrocin- and ferrichrome-type siderophores. In *S. pombe*, the biosynthesis of ferricrocin requires another gene located at the same locus [25], which is also present in *C. fulvum*. However in this latter species, a gene encoding an ABC transporter is located in between the two genes, which might be required for secretion of the predicted siderophore. Iron depletion is known to induce the production of siderophores [50]. Consistent with this, *NPS9* was induced under such condition, but *NPS2* remained barely expressed. Surprisingly, *NPS8* was also induced, suggesting that it might encode a novel type of siderophore synthetase. It is noteworthy that *C. fulvum* does not have any NRPS in the *NPS6* clade that comprises the synthetases responsible for biosynthesis of the external siderophore coprogen. The siderophore system in *C. fulvum* might differ from other fungi such as *C. heterostrophus* and *M. oryzae* by using the putative Nps8 siderophore for iron acquisition or storage. *NPS9* was expressed in runner hyphae of *C. fulvum* prior to penetration of the host and was down-regulated at later stages of infection, suggesting that siderophore production is not crucial for colonization of the host.

Only one core gene, *PKS6*, exhibited high expression under most *in vitro* conditions, consistent with the detection of only cladofuvin as the major compound in liquid shaking cultures. *PKS6* belongs to a gene cluster that is conserved in *Aspergillus* species, which is involved in the production of the related compounds atrochrysone, monodictyphenone and endocrocin. The production of endocrocin anthrone homodimer was reported in *A. oryzae* [32], which shows a structure reminiscent to the one described for cladofulvin. Remarkably, in some *Aspergillus* species, the *mdpH* gene that is missing in *C. fulvum* is involved in directing the biosynthetic pathway towards the production of atrochrysone, and subsequently monodictyphenone, rather than towards the production of endocrocin anthrone [33]. This could explain the similarity in structure between cladofulvin and endocrocin. The two genes of this gene cluster that are specific to *C. fulvum* might be involved in the asymmetrical dimerization of cladofulvin. Altogether, these results suggest that Pks6 is the enzyme responsible for the biosynthesis of cladofulvin, which was the most abundant metabolite detected in all conditions. Functional analysis of *PKS6* is needed to confirm its requirement in cladofulvin production, but attempts to disrupt this gene have been unsuccessful so far.

*PKS1* belongs to a gene cluster homologous to the elsinochrome gene cluster in *E. fawcettii*. All genes except *OXR1* are located within the gene cluster present in *C. fulvum*. The complete elsinochrome biosynthetic pathway has yet to be fully elucidated and there is no function assigned to the hypothetical protein encoded by *OXR1* [43]. It is likely that *C. fulvum* is able to produce elsinochrome or a highly similar compound. Despite its expression in PDB, LC-MS analysis did not detect such a compound. It is possible that the method used to extract SMs was not adapted to the isolation of this type of compound, although this is unlikely because the procedure did extract cladofulvin, which has a chemical structure similar to that of elsinochrome. Functional analysis of *PKS1* will elucidate whether it is responsible for the biosynthesis of an elsinochrome-like compound. Surprisingly, the genome of *C. fulvum* does not contain any gene that belongs to the 1,8-DHN-melanin PKS clade, which suggests that *C. fulvum* is not able to produce DHN-melanin, although it might produce melanin from tyrosine through the DOPA pathway [52]. Alternatively, it was found that a PKS involved in DHN-melanin production in *A. niger* is also responsible for the biosynthesis of several naphtho-γ-pyrones [53]. Similarly, Pks1 could be responsible for the production of elsinochrome and/or DHN-melanin in *C. fulvum*.

### Secondary metabolism and biotrophic growth of *Cladosporium fulvum*

Comparative genomics analyses of necrotrophic and biotrophic fungi suggested that a biotrophic lifestyle is associated with loss of SM genes [2], [6]. Indeed, *Blumeria graminis* and *Tuber melanosporum*, two unrelated biotrophic fungi (an obligate biotrophic pathogen of barley and a symbiont, respectively), share common genomic features: a large genome size due to invasion of transposable elements and a significant reduction of the total number of genes, including SM genes [6], [54]. Sequencing of two rust pathogens, *Melampsora larici-populina* and *Puccinia graminis f. sp. tritici*, revealed two large genomes, but did not show any reduction in gene number [55]. On the contrary, lineage-specific gene families are expanded in these fungi, but surprisingly their genomes contain only one SM core gene for the biosynthesis of a non-ribosomal peptide. These features are similar to those observed in the symbiont *Laccaria bicolor*, which shows expansion of gene families but not of PKS and NRPS gene families [2].

The biotrophic pathogen *C. fulvum* has a large genome size consistent with all these biotrophic fungal species, but does not show any reduction in gene number and has a higher SM production potential than some hemi-biotrophic or necrotrophic fungi [12]. However, EST sequencing and quantitative PCR analyses showed that, apart from *PKS6*, most of the SM genes have a low level of expression *in vitro*. Remarkably, expression levels are even lower during colonization of tomato, including expression of *PKS6*. This gene is highly expressed again when the fungus starts to produce conidiophores and sporulates on the leaf surface. Induction of gene expression at this stage suggests a role for the corresponding SM in survival of the fungus outside its host plant. In accordance with the expression data, only one major compound could be detected and no phytotoxic activity could be observed for SM extracts. Altogether, these data suggest that most SM biosynthetic pathways in *C. fulvum* are cryptic and that low expression and down-regulation of SM gene clusters during infection of its host might be novel mechanisms associated with fungal biotrophy. In addition, pseudogenization appears to have inactivated several core genes and the dothistromin pathway in *C. fulvum* [12], [41]. Likely, *C. fulvum*'s cryptic SM pathways are induced at specific stages of its lifecycle outside its host when the fungus has to survive on the leaf surface or as a saprophyte on dead tissues. Further research is required to determine which environmental or growth conditions can activate these cryptic pathways.

## Supporting Information

File S1 **Supporting information.** Fig. S1. Domain organization of *Cladosporium fulvum* polyketide synthases and non-ribosomal peptide synthetases. Fig. S2. Phylogenetic analysis of *Cladosporium fulvum* PKS and hybrid PKS-NRPS enzymes. Fig. S3. Locus organization of polyketide synthase and hybrid genes in the *Cladosporium fulvum* genome. Fig. S4. Locus organization of non-ribosomal peptide synthetase genes in the *Cladosporium fulvum* genome. Fig. S5. Localization of secondary metabolism genes on chromosomes of *Cladosporium fulvum*. Fig. S6. Expression profile of *Cladosporium fulvum* secondary metabolism functional core genes. Fig. S7. Expression profile of genes located at the *PKS6* gene locus. Fig. S8. Thin layer chromatography (TLC) analysis of *Cladosporium fulvum* secondary metabolites. Fig. S9. Toxicity of cladofulvin on Solanaceous plants. Table S1. Accession number of protein sequences used in phylogenetic analyses. Table S2. Oligonucleotides used in this study. Table S3. ^1^H and ^13^C NMR data for cladofulvin. Table S4. Functional annotation of *Cladosporium fulvum* core secondary metabolism enzymes.
(PDF)
