# Supplementary material for: Secondary Metabolism and Biotrophic Lifestyle in the Tomato Pathogen Cladosporium fulvum
Source: PLoS One. 2014 Jan 17;9(1):e85877. doi: 10.1371/journal.pone.0085877 (PMC3895014; doi:10.1371/journal.pone.0085877)
Supplement: File S1 — Supporting information. Fig. S1. Domain organization of Cladosporium fulvum polyketide synthases and non-ribosomal peptide synthetases. Fig. S2. Phylogenetic analysis of Cladosporium fulvum PKS and hybrid PKS-NRPS enzymes. Fig. S3. Locus organization of polyketide synthase and hybrid genes in the Cladosporium fulvum genome. Fig. S4. Locus organization of non-ribosomal peptide synthetase genes in the Cladosporium fulvum genome. Fig. S5. Localization of secondary metabolism genes on chromosomes of Cladosporium fulvum. Fig. S6. Expression profile of Cladosporium fulvum secondary metabolism functional core genes. Fig. S7. Expression profile of genes located at the PKS6 gene locus. Fig. S8. Thin layer chromatography (TLC) analysis of Cladosporium fulvum secondary metabolites. Fig. S9. Toxicity of cladofulvin on Solanaceous plants. Table S1. Accession number of protein sequences used in phylogenetic analyses. Table S2. Oligonucleotides used in this study. Table S3. 1H and 13C NMR data for cladofulvin. Table S4. Functional annotation of Cladosporium fulvum core secondary metabolism enzymes. (PDF) [file pone.0085877.s001.pdf]

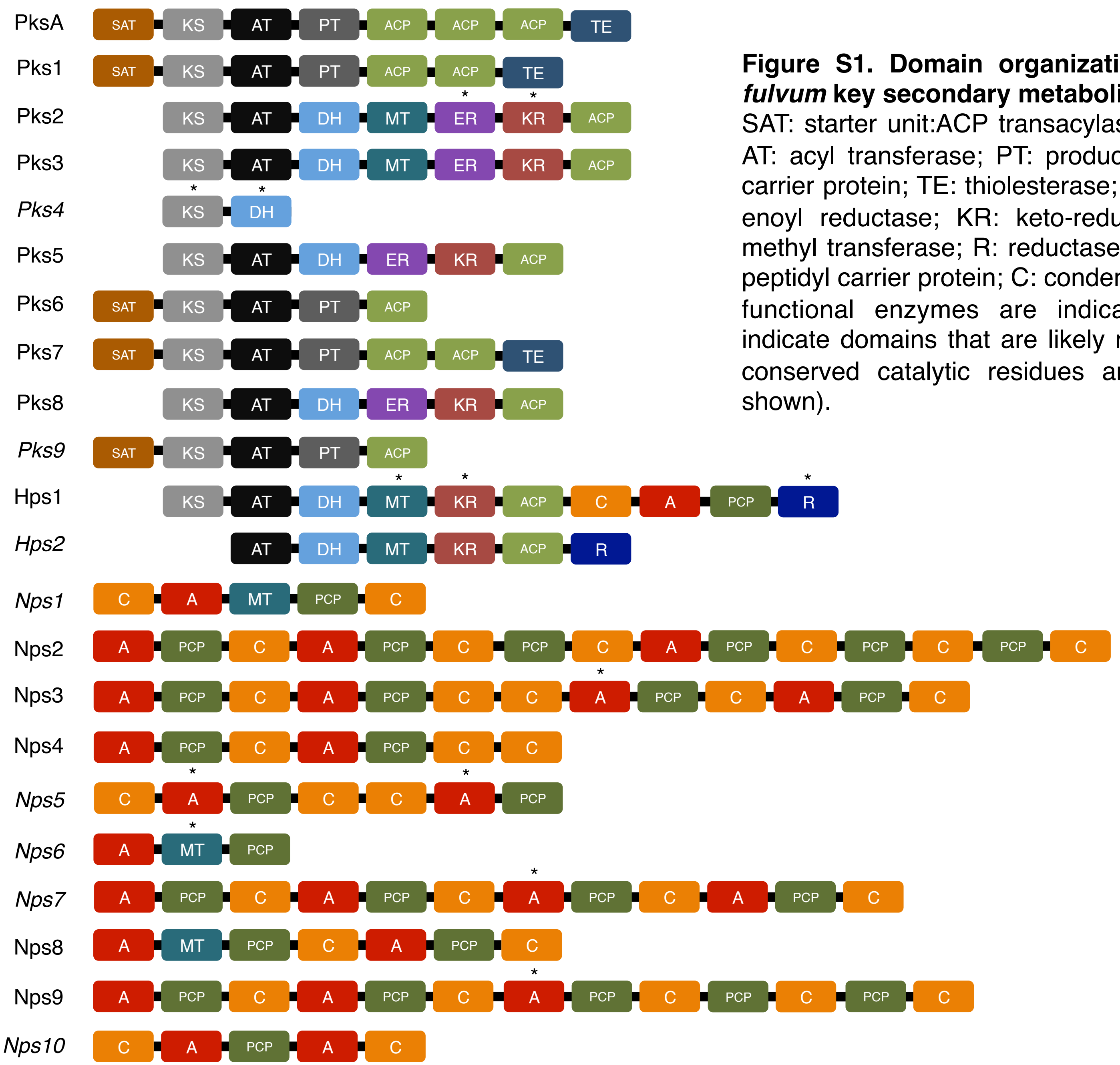

Figure S1. Domain organization of *Cladosporium fulvum* key secondary metabolism enzymes. SAT: starter unit:ACP transacylase; KS: keto-synthase; AT: acyl transferase; PT: product template; ACP: acyl carrier protein; TE: thiolesterase; DH: dehydratase; ER: enoyl reductase; KR: keto-reductase; MT: *C*- or *N*-methyl transferase; R: reductase; A: adenylation; PCP: peptidyl carrier protein; C: condensation. Predicted non-functional enzymes are indicated in italics. Stars indicate domains that are likely not functional because conserved catalytic residues are mutated (data not shown).

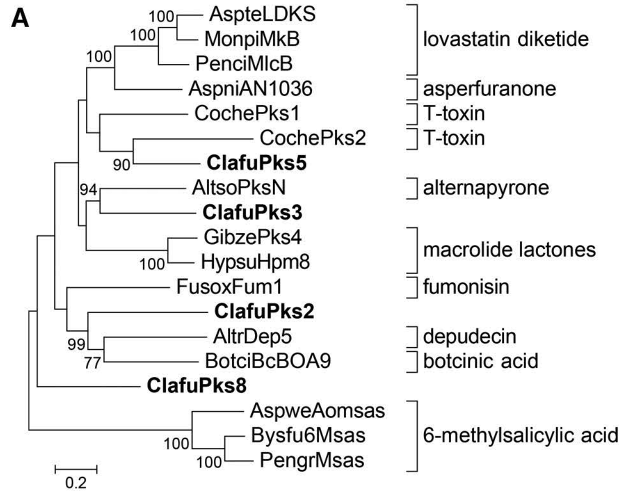

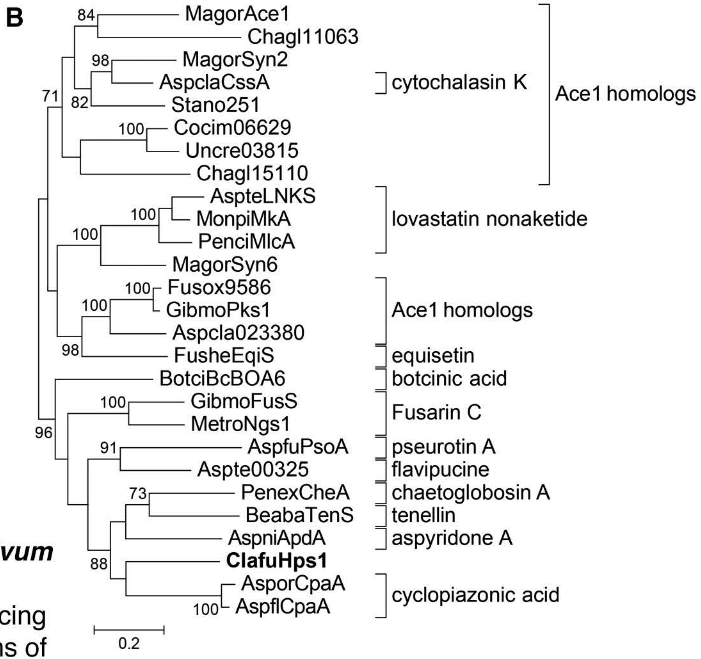

Fig. S2. Phylogenetic analysis of *Cladosporium fulvum* PKS and hybrid PKS-NRPS enzymes.
Alignments of **(a)** full-length protein sequences of reducing polyketide synthases (PKS) and **(b)** KS and AT domains of hybrid polyketide synthase-non-ribosomal peptide synthetases (PKS-NRPS) were used to construct maximum likelihood phylogenetic trees. Only bootstrap values over 70 are shown. *C. fulvum* secondary metabolism enzymes are indicated in bold. Accession numbers are given in Table S1 and Table S4.

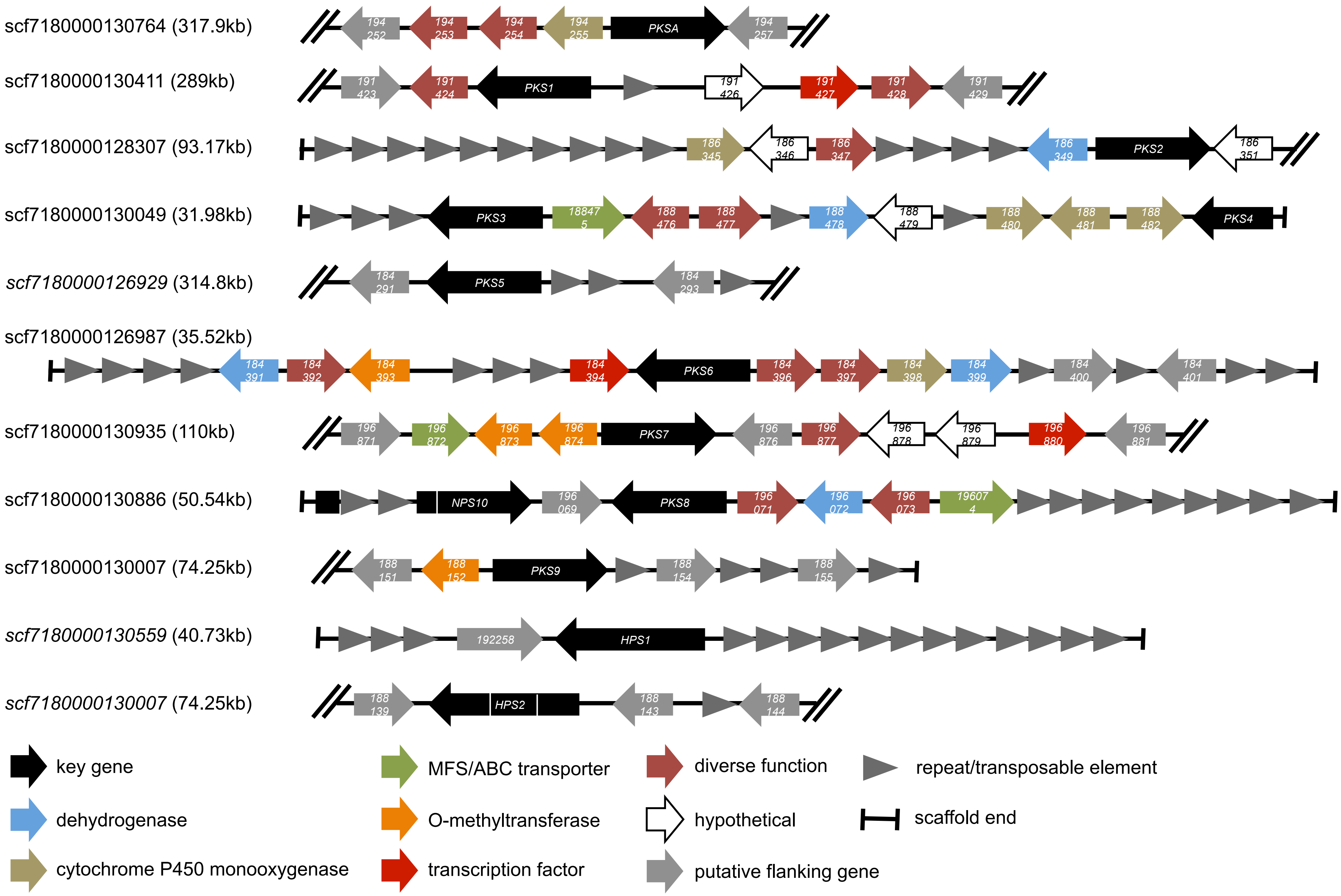

Figure S3. Locus organization of polyketide synthase and hybrid genes in the *Cladosporium fulvum* genome. White lines within key genes indicate pseudogenization. The PKSA gene cluster is fully depicted in De Wit *et al*. (2012) and Chettri *et al*. (2013).

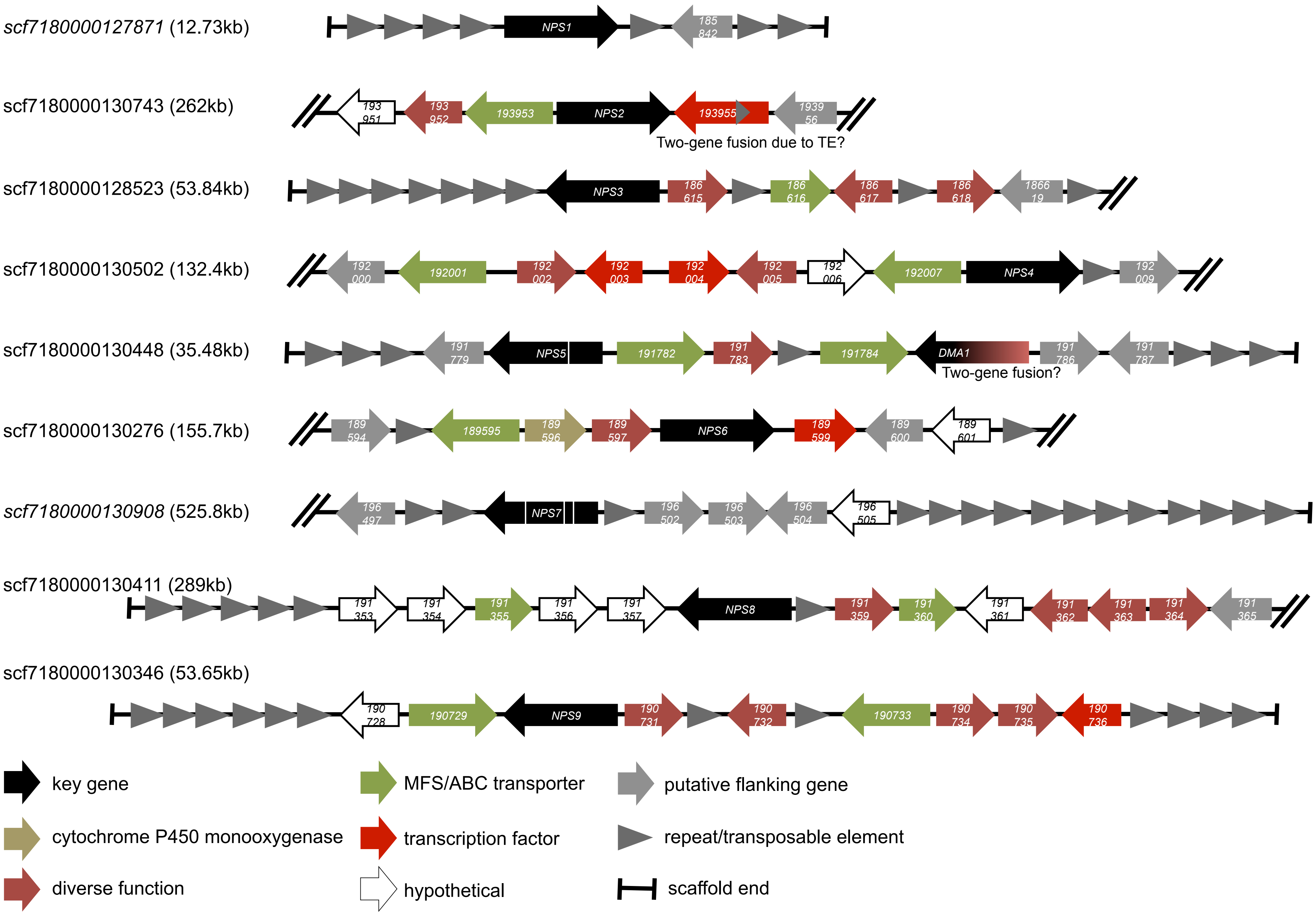

Figure S4. Locus organization of non-ribosomal peptide synthetase genes in the *Cladosporium fulvum* genome. White lines within key genes indicate pseudogenization.

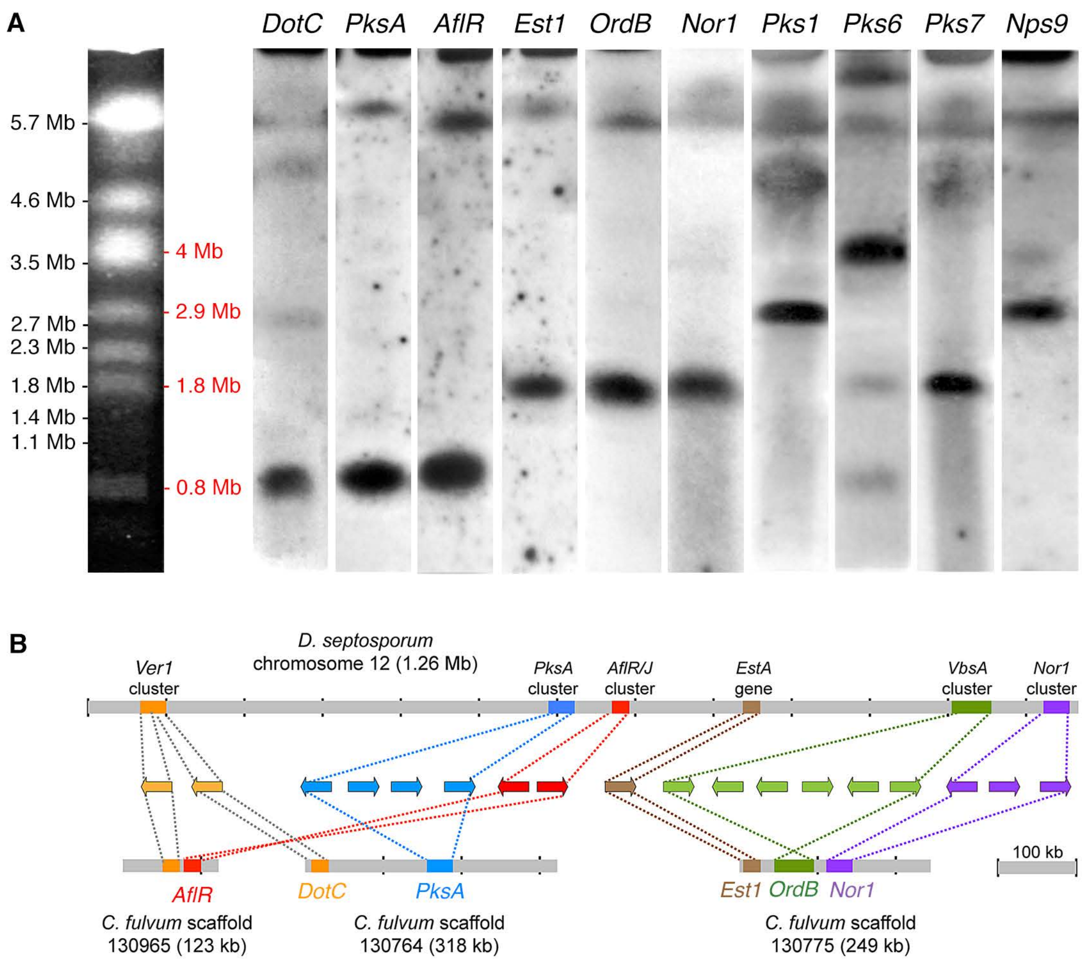

Fig. S5. Localization of secondary metabolism genes on chromosomes of *Cladosporium fulvum*. (a) CHEF gel electrophoresis was performed to separate chromosomes of *C. fulvum* (left lane) and large size DNA was transferred onto nylon membrane. Specific probes corresponding to genes from different clusters were hybridized to identify chromosomes that carry them. Size standards indicated on the left are from chromosome preparations of *Schizosaccharomyces pombe* and *Hansenula wingei*. In red are indicated the size of the *C. fulvum* chromosomes that carry secondary metabolism genes. **(b)** Organization of the dothistromin gene cluster is shown in *Dothistroma septosporum* and *C. fulvum* (adpated from de Wit *et al*., 2012).

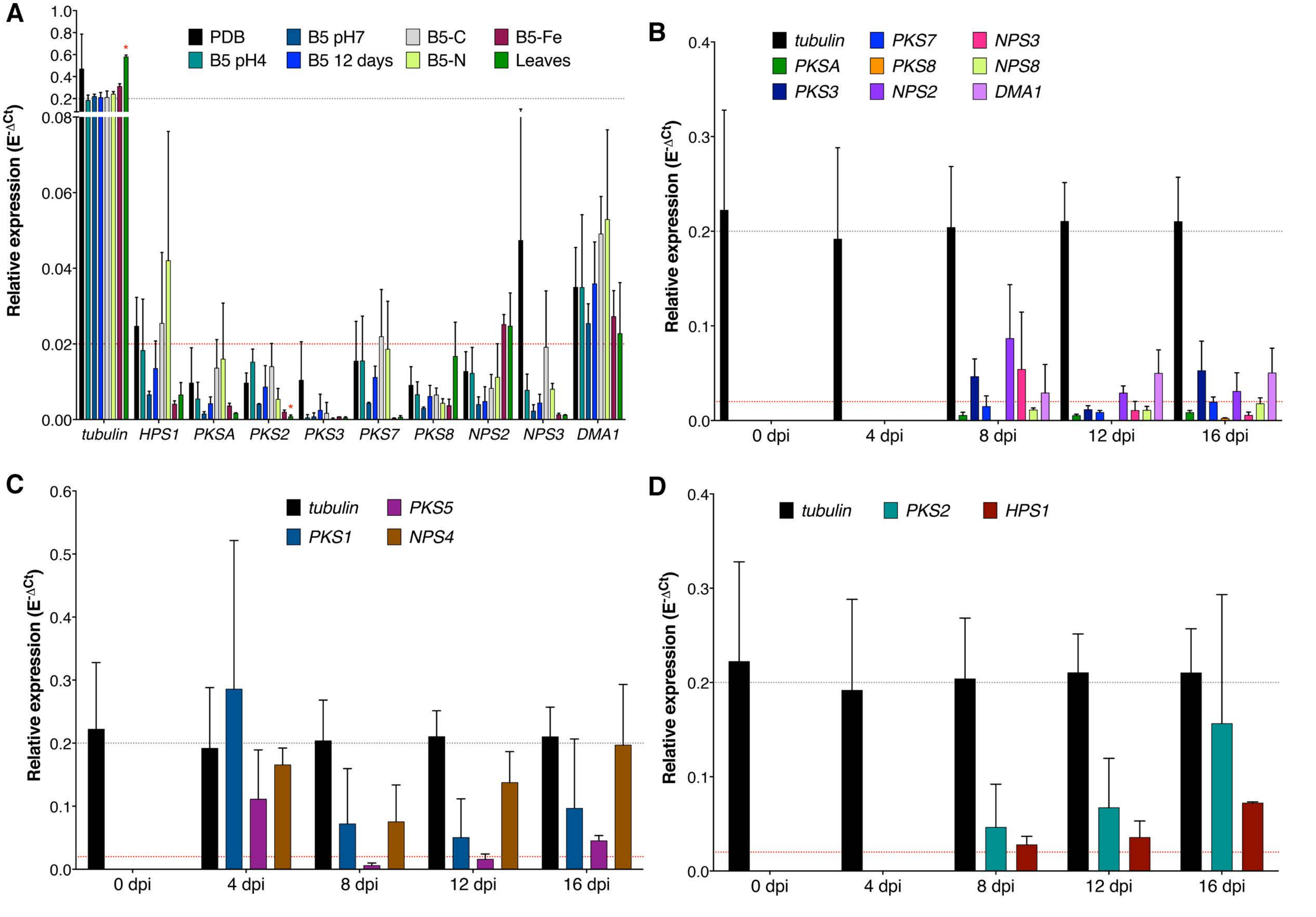

Fig. S6. Expression profile of *Cladosporium fulvum* secondary metabolism functional key genes. Gene expression was measured using RT-qrtPCR **(a)** under *in vitro* conditions, including B5 without carbon (C), nitrogen (N) or iron (Fe), and autoclaved leaves; and **(b)**, **(c)** and **(d)** during infection of tomato from 0 to 16 days post inoculation (dpi). **(c)** Genes for which expression seems to decrease over time. **(d)** Genes for which expression seems to increase over time. The data was normalized to the actin gene and analyzed using the $E^{-\Delta Ct}$ method, where E is the primer pair efficiency of a given gene. The grey dotted line indicates the tubulin expression level and the red dotted lines indicate the expression threshold 10-fold lower than the average tubulin expression level. Values are the mean of three biological repeats and error bars represent the standard deviation. Expression in each *in vitro* condition was compared to expression in B5 pH4 using multiple t-tests , not assuming consistent SD, correcting for multiple comparisons with the Holm-Sidak method, at the alpha significance threshold of 0.05. For each gene, each *in planta* time point was compared to the previous one using a Two-way ANOVA followed by a multiple comparison test corrected with the Holm-Sidak method at the alpha significance threshold of 0.05. Significant differences are indicated by red stars.

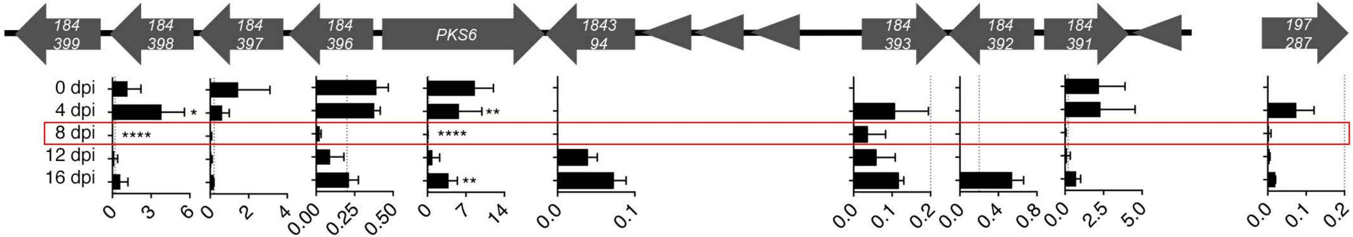

Fig. S7. Expression profile of genes located at the *PKS6* gene locus.
Gene expression was measured using RT-qrtPCR during infection of tomato from 0 to 16 days post inoculation (dpi). The data was normalized to the actin gene and relative expression is indicated on the X axis using the $E^{-\Delta Ct}$ method, where E is the primer pair efficiency of a given gene. The grey dotted line indicates the tubulin expression level. The red square indicates the time point when the expression of genes is at the lowest level.

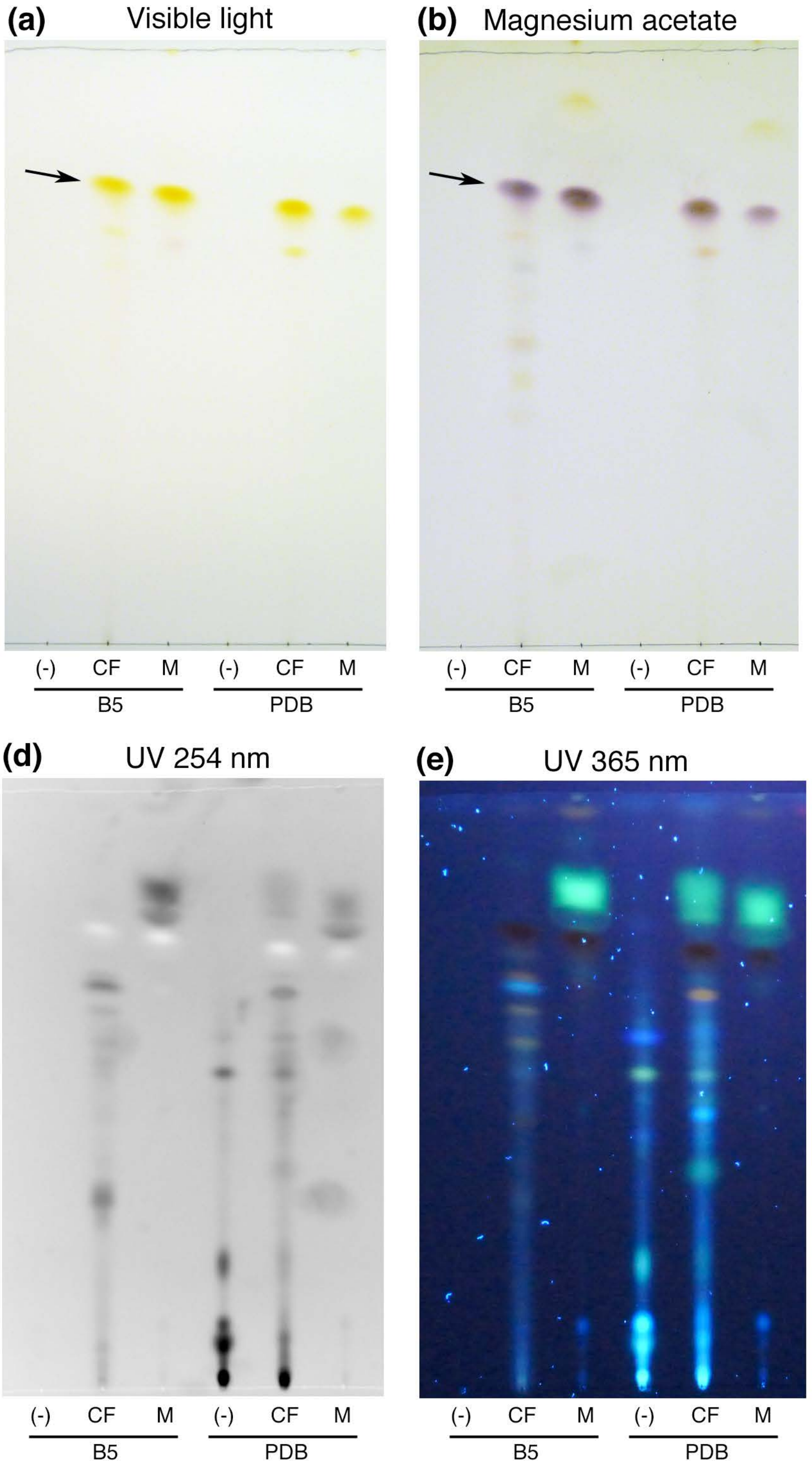

Figure S8. Thin layer chromatography (TLC) analysis of *Cladosporium fulvum* secondary metabolites. TLC plate pictures were taken **(a)** under visible light, **(b)** after staining with 4% magnesium acetate in methanol, **(c)** after iodine staining, **(d)** under 254 nm UV light and **(e)** under 365 nm UV light. Arrows indicate the position of cladofulvin. (-) control (sterile medium); CF, culture filtrate; M, mycelium.

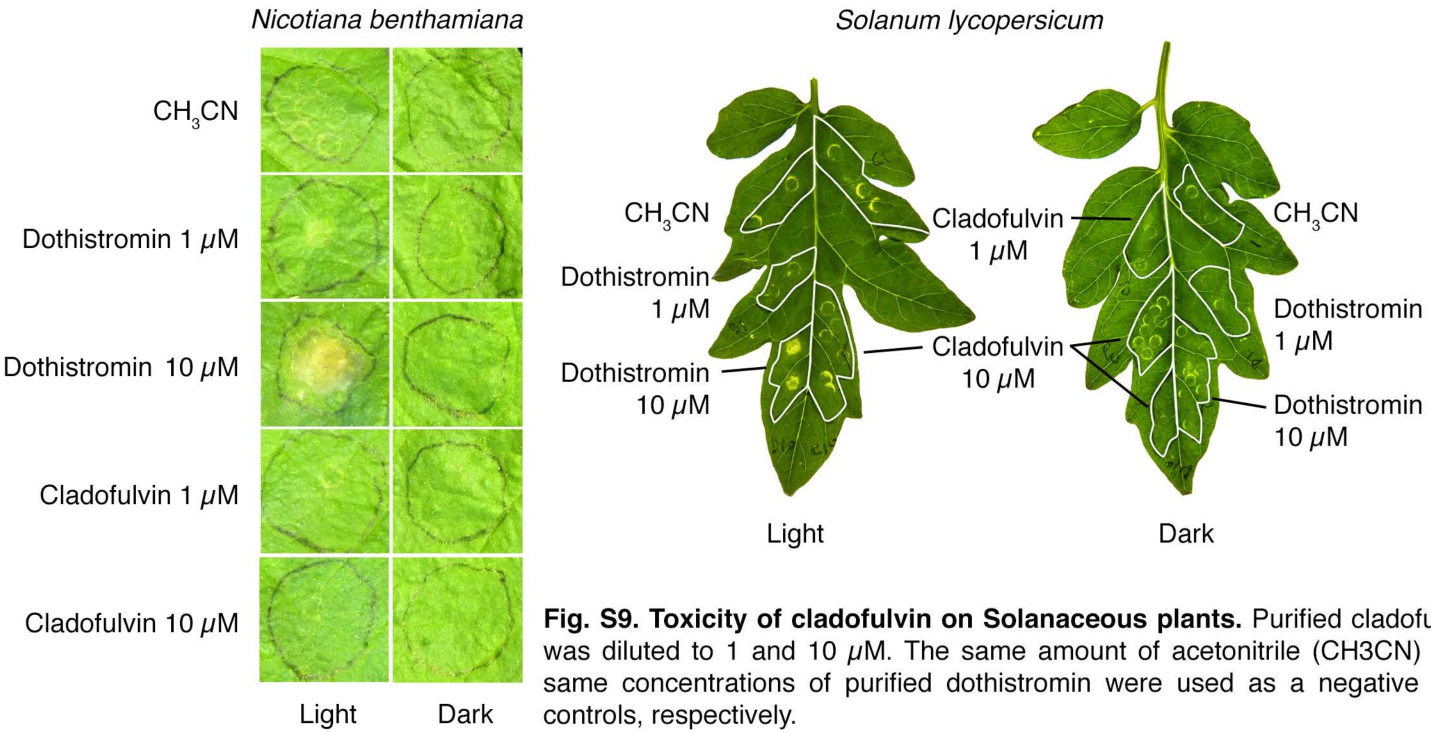

Fig. S9. Toxicity of cladofulvin on Solanaceous plants. Purified cladofulvin was diluted to 1 and 10 μM. The same amount of acetonitrile (CH3CN) and same concentrations of purified dothistromin were used as a negative and controls, respectively.

Table S1. Accession numbers of protein sequences used in phylogenetic analyses.

Core enzyme	Fungal species	GenBank accession number
SormaPks	*Sordaria macrospora*	CAM35471.1
PodanPks1	*Podospora anserina*	XP_001910795.1
MagorAlb1	*Magnaporthe oryzae*	XP_003715434.1
NodspPks1	*Nodulisporium sp. ATCC74245*	AAD38786.1
CollaPks1	*Colletotrichum lagenaria*	BAA18956.1
GlaloPks1	*Glarea lozoyensis*	AAN59953.1
ElsfaPks1	*Elsinoe fawcettii*	ABU63483.1
WandePks1	*Wangiella dermatidis*	AAD31436.3
DotsePksA	*Dothistroma septosporum*	EME39092.1
AspniStcA	*Aspergillus nidulans*	AAC49191.1
AspflPksA	*Aspergillus flavus*	AAS90093.1
CerniCtb1	*Cercospora nicotianae*	AAT69682.1
NechaPKSN	*Nectria haematococca*	AAS48892.1
AspniYwa1	*Aspergillus nidulans*	CAA46695.2
AspniAlbA	*Aspergillus niger*	EHA28527.1
AspfuAlb1	*Aspergillus fumigatus*	AAC39471.1
GibzePks12	*Gibberella zeae*	AAU10633.1
GibfuBik1	*Gibberella fujikuroi*	CAB92399.1
AspteAcas	*Aspergillus terreus*	XP_001217072.1
AspniMdpG	*Aspergillus nidulans*	XP_657754.1
AspfuEncA	*Aspergillus fumigatus*	XP_746435.1
GibzePks13	*Gibberella zeae*	ABB90282.1
HypsuHpm3	*Hypomyces subiculosus*	ACD39762.1
MonpuPksCT	*Monascus purpureus*	BAD44749.1
AspniAN1034	*Aspergillus nidulans*	EAA65602.1
PenbrMpaC	*Penicillium brevicompactum*	ADY00130.1
GibzeFsl1	*Gibberella zeae*	XP_390640.1
CocheNps1	*Cochliobolus heterostrophus*	AAX09983.1
CocheNps3	*Cochliobolus heterostrophus*	AAX09985.1
FuseqEsyn1	*Fusarium equiseti*	Q00869.2
UstmaSid2	*Ustilago maydis*	AAB93493.1
MagorSsm1	*Magnaporthe oryzae*	XP_003719607.1
CocheNps2	*Cochliobolus heterostrophus*	AAX09984.1
SchpoSib1	*Schizosaccharomyces pombe*	CAB72227.1
AspfuSidC	*Aspergillus fumigatus*	XP_753088.1
UstmaFer3	*Ustilago maydis*	DAA04939.1
OmpolFso1	*Omphalotus olearius*	AAX49356.1
TriviTex1	*Trichoderma virens*	AAM78457.1
ClapuLpsA1	*Claviceps purpurea*	AET79183.1
CocheNps4	*Cochliobolus heterostrophus*	AAX09986.1
AspfuPes1	*Aspergillus fumigatus*	XP_752404.1
CoccaHts1	*Cochliobolus carbonum*	Q01886.2
AspfuPesL	*Aspergillus fumigatus*	XP_751084.1
AltalAmt	*Alternaria alternata*	AAF01762.1
CocheNps5	*Cochliobolus heterostrophus*	AAX09987.1
MagorSsm2	*Magnaporthe oryzae*	XP_003714007.1
GibzeNps6	*Gibberella zeae*	XP_383923.1
CocheNPS6	*Cochliobolus heterostrophus*	AAX09988.1

Table S1. Accession numbers of protein sequences used in phylogenetic analyses (continued).

Core enzyme	Fungal species	GenBank accession number
AspfuSidD	*Aspergillus fumigatus*	XP_748662.1
CocheNps8	*Cochliobolus heterostrophus*	AAX09990.1
EpifePerA	*Epichloe festucae*	BAE06845.2
AspteLDKS	*Aspergillus terreus*	AAD34559.1
MonpiMkB	*Monascus pilosus*	ABA02240.1
PenciMlcB	*Penicillium citrinum*	BAC20566.1
AspniAN1036	*Aspergillus nidulans*	EAA65604.1
CochePks1	*Cochliobolus heterostrophus*	AAB08104.3
CochePks2	*Cochliobolus heterostrophus*	ABB76806.1
AltsoPksN	*Alternaria solani*	BAD83684.1
GibzePks4	*Gibberella zeae*	ABB90283.1
HypsuHpm8	*Hypomyces subiculosus*	ACD39767.1
FusoxFum1	*Fusarium oxysporum*	ACB12550.1
AltbrDep5	*Alternaria brassicicola*	ACZ57548.1
BotciBcBOA9	*Botrytis cinerea*	CBX87032.1
AspweAomsas	*Aspergillus westerdijkiae*	AAS98200.1
Bysni6Msas	*Byssochlamys nivea*	AAK48943.1
PengrMsas	*Penicillium griseofulvum*	P22367.1
MagorAce1	*Magnaporthe oryzae*	CAG28797.1
Chagl11063	*Chaetomium globosum*	XP_001220460.1
MagorSyn2	*Magnaporthe oryzae*	CAG28798.1
AspclaCssA	*Aspergillus clavatus*	XP_001270543.1
Stano251	*Stagonospora nodorum*	XP_001790998.1
Cocim06629	*Coccidioides imitis*	XP_001242733.1
Uncre03815	*Uncinocarpus reesii*	EEP78969.1
Chagl15110	*Chaetomium globosum*	XP_001221381.1
AspteLNKS	*Aspergillus terreus*	Q9Y8A5.1
MonpiMkA	*Monascus pilosus*	ABA02239.1
PenciMlcA	*Penicillium citrinum*	BAC20564.1
MagorSyn6	*Magnaporthe oryzae*	CAG29113.1
Fusox9586	*Fusarium oxysporum*	EGU88865.1
GibmoPks1	*Gibberella moniliformis*	AAR92208.1
Aspcla023380	*Aspergillus clavatus*	XP_001269050.1
FusheEqiS	*Fusarium heterosporum*	AGO86662.1
BotciBcBOA6	*Botrytis cinerea*	CAP58786.1
GibmoFusS	*Gibberella moniliformis*	AAT28740.1
MetroNgs1	*Metarhizium robertsii*	ACS68554.1
AspfuPsoA	*Aspergillus fumigatus*	ABS87601.1
Aspte00325	*Aspergillus terreus*	EAU38971.1
PenexCheA	*Penicillium expansum*	CAO91861.1
BeabaTenS	*Beauveria bassiana*	CAL69597.1
AspniApdA	*Aspergillus nidulans*	XP_681681.1
AsporCpaA	*Aspergillus oryzae*	BAK26562.1
AspflCpaA	*Aspergillus flavus*	BAI43678.1

Table S2. Oligonucleotides used in this study.

Name	Sequence (5′ to 3′)	Name	Sequence (5′ to 3′)
qHPS1_F	CTTGCCAGTGGGTCTACCAT	*q197287_F*	AGATCCGGCGTGAATACAAC
qHPS1_R	TAGGATCACTTCGCCTGCTT	*q197287_R*	TTCCTGCCAGCTTGACTTTT
qPKSA_F	TCCCGGCTCAGATTGATAAC	*q184391_F*	AGCTTCGGTCATCTCAAGGA
qPKSA_R	CCGACGTATAGAGGCTGCTC	*q184391_R*	CGAGTGTCGAGGAACACTGA
qPKS1_F	GTGATGCACTGAAGGCTCAA	*q184392_F*	CAGTTCCAAAGCCTGCCTAC
qPKS1_R	AGCAAGTTGGTCGAGCTGAT	*q184392_R*	AGGAAGTGTGGACTGGATGC
qPKS2_F	TGTGGCTATTGCACTCGAAG	*q184393_F*	GTGGTGGATTTCAGCCTGTT
qPKS2_R	TCCATTGATCTGATGCCGTA	*q184393_R*	TCATCGACGATTGTGGTGTT
qPKS3_F	GCGTAGGTCAGGCTGCTATC	*q184394_F*	TCTGTCTAGACGGCGAGGAT
qPKS3_R	CGAGTGAGTTGAGGACGACA	*q184394_R*	CTTCGAAGATCCGTTTCGAG
qPKS5_F	TGCTGGTATCGTGGGTAACA	*q184396_F*	GGACCTGGAGCATCACATCT
qPKS5_R	CAGAGTTCTCGGCCAGGTAG	*q184396_R*	GGATGGTGTCACCCGTAAAC
qPKS6_F	CTGCATATCGGAGCAGTGAA	*q184397_F*	CTTGTCAAGGTCATGCGAGA
qPKS6_R	TTGCGTTTCTTGAAGTCGTG	*q184397_R*	GGCAGATCGCTTGAGTATCC
qPKS7_F	AGCTGAAGAACGGAAGTGGA	*q184398_F*	GATGATCGGACACTGGACCT
qPKS7_R	GTGTTCTGTTGGGCACAATG	*q184398_R*	CACACCCAAAGGCGTAAGAT
qPKS8_F	TGACATCGCAGACTTCCTTG	*q184401_F*	AACAGCAGAAAGGACGGAAA
qPKS8_R	AGGGCAAAGGAAGCGATATT	*q184401_R*	GAGTTCCTGGGTTCCTCTCC
qNPS2_F	TGGACTCACAGCGCACTATC	*q184402_F*	AACAGCAGAAAGGACGGAAA
qNPS2_R	ATACGGACGGTCTTGTCTGG	*q184402_R*	GAGTTCCTGGGTTCCTCTCC
qNPS3_F	GTACACTTGTGGCGGATGTG		
qNPS3_R	TCATGTACGCTGGAAGCAAG		
qNPS4_F	GTGGCTCTAGCGGCATACTC		
qNPS4_R	TCCTGCCAGTAGCTGGTCTT		
qNPS6_F	AATGGCTAAAACACGCCATC		
qNPS6_R	TCCAACGAATTCCAGACTCC		
qNPS8_F	ACTCTCTTCGTTTGGCAGGA		
qNPS8_R	CTCATGAGCTTGCGTTGGTA		
qNPS9_F	ATATGGCCCGACTCACTACG		
qNPS9_R	CAGTGAGCGATTCTGTTGGA		
qDMA1_F	TGTGCTGGTACTGCCTTCAG		
qDMA1_R	CCGTCTTGTTGACATGTTGC		

Oligonucleotides used to measure *actin*, *tubulin*, *Avr4* and *Avr9* gene expression are the same as in de Wit *et al.* (2012).

Table S3. ^{1}H and ^{13}C NMR data for cladofulvin.

The NMR data collected for the purified compound is identical to the values reported for cladofulvin in the literature.

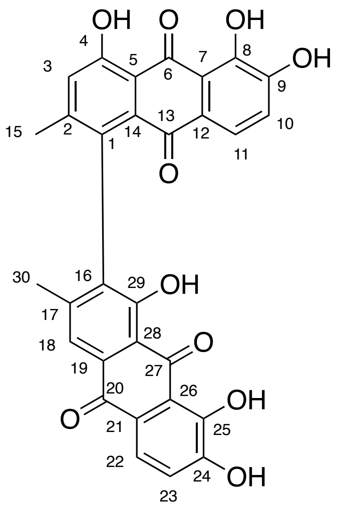

Position	δC	δH
1	129.3	-
2	149.3	-
3	124.7	7.35, s, 1H
4	162.3	-
5	115.2	-
6	181.4	-
7	115.9	-
8	149.8	-
9	152.1	-
10	121.1	7.11, d, 1H, J = 8.2 Hz
11	121.4	7.46, d, 1H, J = 8.2 Hz
12	125.1	-
13	193.3	-
14	131.3	-
15	19.6	2.03, s, 3H
16	136.0	-
17	146.3	-
18	121.5	7.77, s, 1H, J = 8.1 Hz
19	132.3	-
20	180.9	-
21	124.9	-
22	121.4 (2xC)	7.76, d, 1H
23	120.9	7.21, d, 1H, J = 8.1 Hz
24	150.4	-
25	152.3	-
26	116.4	-
27	193.4	-
28	114.1	-
29	162.5	-
30	19.5	2.02, s, 3H

Table S4. Functional annotation of *Cladosporium fulvum* key secondary metabolism enzymes.

Gene	Protein ID[a]	Best BlastP hit (NCBI)[b]	e-value	IPS domains[c]	IGS domains[c]	ASMPKS domains[c]	Predicted precursor[d]
PKSA	194256	AAZ95017.1 polyketide synthase *Mycosphaerella pini*	0.0	KS-AT-ACP-ACP-ACP-TE	KS-AT-?-ACP-ACP-ACP-TE	KS-AT-ACP-ACP-ACP-TE	Malonyl-CoA
PKS1	191425	EFQ92987.1 hypothetical protein PTT_09773 *Pyrenophora teres*	0.0	KS-AT-ACP-ACP-TE	KS-AT-?-ACP-ACP-TE	KA-AT-ACP-ACP-TE	Malonyl-CoA
PKS2	186350	CBI52337.1 putative polyketide synthase *Sordaria macrospora*	0.0	KS-AT-ME-ER-KR-ACP	KS-AT-DH-?-?-KR-ACP	KS-AT-DH-KR-ACP	Methylmalonyl-CoA
PKS3	188474	AAR90260.1 polyketide synthase *Cochliobolus heterostrophus*	0.0	KS-AT-ME-ER-KR-ACP	KS-AT-DH-?-ER-KR	KS-AT-DH-ER-KR	Malonyl-CoA
PKS4	188483	XP_002482968.1 putative polyketide synthase *Talaromyces stipitatus*	1e-62	KS-DH	none	none	n.d.
PKS5	184292	XP_003042842.1 hypothetical NECHADRAFT_106474 *Nectria haematococca*	0.0	KS-AT-ER-KR-ACP	KS-AT-?-ER-KR	KS-AT-DH-ER-KR-ACP	Malonyl-CoA
PKS6	184395	XP_002482968.1 putative polyketide synthase *Talaromyces stipitatus*	0.0	KS-AT-ACP	KS-AT-?-ACP	KS-AT-ACP	Malonyl-CoA
PKS7	196875	ADO14690.1 cercosporin polyketide synthase *Mycosphaerella coffeicola*	0.0	KS-AT-ACP-ACP-TE	KS-AT-ACP-ACP-TE	KS-AT-ACP-ACP-TE	Malonyl-CoA
PKS8	196070	AAR90246.1 PKS10 *Botryotinia fuckeliana*	0.0	KS-AT-ER-KR-ACP	?-?-ER-KR-ACP	KS-AT-DH-ER-KR-ACP	Methylmalonyl-CoA
PKS9	188153	XP_657754.1 hypothetical protein AN0150.2 *Aspergillus nidulans*	0.0	KS-AT-ACP	KS-?-ACP	KS-AT-ACP	Malonyl-CoA
HPS1	192259	XP_002486604.1 putative PKS-NRPS *Talaromyces stipitatus*	0.0	KS-AT-ME-KR-ACP-C-A-PCP	KS-AT-?-?-KR-?-A-PCP	KS-AT-DH-KR-ACP	Malonyl-CoA / DMAFASVI new signature
HPS2	188140 / 188141 / 188142	XP_002153037.1 PMAA_009380 *Penicillium marneffei*	0.0	KS-AT-ME-KR-ACP	?-?-KR-ACP	AT-DH-KR-ACP	Malonyl-CoA
NPS1	185841	ACJ04424.1 aureobasidin A1 biosynthesis complex *Aureobasidium pullulans*	0.0	C-A-PCP-C	?-A-ME-PCP	-	DAWLYVAV CssA-M1-D-Ala|Cyclosporine synthetase CssA Val
NPS2	193954	AAD00581.2 peptide synthetase *Aureobasidium pullulans*	0.0	A-PCP-C-A-PCP-C-PCP-C-A-PCP-C-PCP-C-PCP-C	A1-PCP-?-A2-PCP-?-PCP-?-A3-PCP-?-PCP-?-PCP-?	-	A1: DVFELIMI Gly / A2: DVFSVAXX Ser/Ala / A3: DVLDIGGI N^5-hydroxy-N^5-acetyl-L-Orn
NPS3	186614	XP_002380231.1 putative NRPS *Aspergillus flavus*	0.0	A-PCP-C-A-PCP-C-C-A-PCP-C-A-PCP-C	A1-PCP-?-A2-PCP-?-?-PCP-?-A3-PCP	-	A1: DVWNLSTF EntF/SyrE Ser / A2: DVICVA-V new signature / A3: DVSYAGXX new signature
NPS4	192008	EFY94582.1 NRPS *Metarhizium anisopliae*	0.0	A-PCP-C-A-PCP-C-C	A1-PCP-?-A2-PCP-?	-	A1: DASDIAVP new signature / A2: DVSDVGPP new signature
NPS5	191780 / 191781	XP_002843341.1 NRPS *Arthroderma otae*	0.0	C-A-PCP-C-C-A-PCP	?-PCP-?-?-PCP	-	n.d.

Table S4. Functional annotation of *Cladosporium fulvum* key secondary metabolism enzymes (continued).

Gene	Protein ID[a]	Best BlastP hit (NCBI)[b]	e-value	IPS domains[c]	IGS domains[c]	ASMPKS domains[c]	Predicted precursor[d]
NPS6	189598	XP_001267502.1 putative NRPS *Neosartorya fischeri*	8e-141	A-PCP	A-?-PCP	-	A: DAGDIGFP new signature
NPS7	196498 / 196499 / 196500 / 196501	Q01886.2 HC-toxin synthetase *Cochliobolus carbonum*	2e-84	A-C-A-A	A1-?-A2-A3	-	A1: DASFVIGF new signature / A2: DNQ-VGAI new signature / A3: DVYARGXX new signature
NPS8	191358	AAX09983.1 NPS1 *Cochliobolus heterostrophus*	0.0	A-PCP-C-A-PCP-C	A1-ME-PCP-?-A2-PCP	-	A1: DAMVVGGV TycB-M2-L-Phe/L-Trp|tyrocidine synthetase / A2: DGFFEGIP BacA/FenB Ile
NPS9	190730	XP_001821068.1 SidC *Aspergillus oryzae*	0.0	A-PCP-C-A-PCP-C-A-PCP-C-PCP-C-PCP-C	A1-PCP-?-A2-PCP-?-A3-PCP-?-PCP-?-PCP-?	-	A1: DPMMWMAI Ser / A2: DVQHTITV Gly / A3: DVGSSGAI N^5-hydroxy-N^5-acetyl-L-Orn
NPS10	196066 / 196067 / 196068	XP_002486632.1 putative aminoadipate-semialdehyde dehydrogenase *Talaromyces stipitatus*	2e-56	C-A-PCP-A-C	PCP	-	n.d.
DMA1	191785	XP_003298803.1 hypothetical protein PTT_09620 *Pyrenophora teres f. teres*	3e-128	IPR017795 Aromatic prenyltransferase, DMATS type	-	-	-

[a] Protein ID from the Doe Joint Genome Institute resource (www.jgi.doe.gov).

[b] BlastP was performed using the non-redundant protein database of NCBI (www.ncbi.nlm.nih.gov).

[c] Search for conserved domains was carried out with InterproScan (IPS; www.ebi.ac.uk), PKS/NRPS analysis website (IGS; nrps.igs.umaryland.edu) and ASMPKS (gate.smallsoft.co.kr:8008/~hstae/asmpks/pks_prediction.pl). KS: keto-synthase; AT: acyl transferase; DH: dehydratase; ER: enoyl reductase; KR: keto-reductase; ME: methyl transferase; ACP: acyl carrier protein; TE: thio-esterase; A: adenylation; C: condensation; PCP: peptidyl carrier protein.

[d] Prediction made by ASMPKS for acyl specificity and by PKS/NRPS analysis website for amino acid specificity. Signatures of A domains were also compared to those described for siderophore synthetases and bacterial synthetases. n.d.: not determined.
